# Biochemical and structural characterization of a tail-spike protein with depolymerase activity identified in a marine podovirus

**DOI:** 10.1107/S2059798326005425

**Published:** 2026-06-17

**Authors:** Serena Sirigu, Thomas Roret, Pierre-Yves Mocaër, Robert Larocque, Diane Jouanneau, Pierre Legrand, Anne-Claire Baudoux, Mirjam Czjzek

**Affiliations:** a Synchrotron SOLEIL L’Orme des Merisiers, Départementale 128 91190Saint-Aubin France; b Sorbonne Université, CNRS, UMR 8227, LBI2M, Station Biologique de Roscoff 29680Roscoff France; c Sorbonne Université, CNRS, UMR 7144, Station Biologique de Roscoff 29680Roscoff France; University of Alabama at Birmingham, USA

**Keywords:** marine phages, podoviruses, EPS depolymerases, tail-spike proteins, crystal structure, SAXS

## Abstract

Marine phages are, through the infection of their bacterial hosts, key regulators of microbiome and carbon fluxes in the ocean. Despite their important role, the specific molecular mechanisms that underlie infection are so far understudied. Previously, the podovirus *Cobetia marina* virus 1 (Carin-1), which infects the marine γ-proteobacterium *C. marina*, was shown to display exopolysaccharide depolymerase activity. This activity is likely to mediate degradation of the host capsule to facilitate access to the bacterial membrane receptor, but no corresponding gene could be annotated in the genome of Carin-1 by comparative genomics. Biochemical characterization enabled assignment of this activity to Dpo31, a protein sharing less than 10% sequence identity with any characterized protein. Here, we report the structural domain organization and biochemical characterization of Dpo31, revealing an overall structure that is analogous to podovirus tail-spike proteins, allowing us to locate the depolymerase activity to the D3 domain and to identify original structural features that explain the absence of detectable similarity at the primary-sequence level.

## Abstract 2

The marine tail-spike protein Dpo31 degrades the exopolysaccharide of its host and has structural features similar to those of other members of this protein class, despite similarity not being detected at the sequence level.

## 1. Introduction

The tail structure of bacterial viruses (bacteriophages) that belong to the class *Caudoviricetes* (previously known as *Caudoviridae*) is a determinant of host recognition and infection (Labrie *et al.*, 2010 ▸; Leiman & Molineux, 2008 ▸). The tail fibers or tail-spike proteins commonly exhibit depolymerases (Dpos) that are involved in the early steps of infection. These enzymes facilitate the binding and degradation of bacterial host extracellular polysaccharides [capsular polysaccharides (CPS), exopolysaccharides (EPS) or lipopolysaccharide (LPS)] in order to access the bacterial cell wall for DNA injection (Sutherland, 1995 ▸, 1999 ▸; Latka *et al.*, 2017 ▸). Dpos fall into two main enzymatic classes of polysaccharidases, hydrolases and lyases (the latter constituting a large majority of well characterized and experimentally validated depolymerases), that act endolytically, cleaving specific internal glycosidic bonds at multiple positions along the polymer chain (Sutherland, 1995 ▸, 1999 ▸; Latka *et al.*, 2017 ▸; Knecht *et al.*, 2020 ▸). Despite their common biological role, Dpos display a high level of functional and molecular diversity and target various substrates (Latka *et al.*, 2017 ▸; Knecht *et al.*, 2020 ▸). To our knowledge, studies on virus-derived Dpos have largely focused on short-tailed podoviruses that infect pathogenic and biofilm-forming bacteria (Cornelissen *et al.*, 2011 ▸, 2012 ▸; Jia *et al.*, 2025 ▸). More recently, viruses of marine bacteria were also shown to possess depolymerases active against EPS released by their hosts (Lelchat *et al.*, 2019 ▸).

EPS derived from marine bacteria consist mostly of polysaccharides, but also proteins, lipids and DNA. The polysaccharidic moiety displays a wide chemical diversity with homopolymeric or heteropolymeric composition, including numerous rare and specific monosaccharides, linear or branched backbones, and inorganic or organic substituents (Decho & Gutierrez, 2017 ▸; Vincent *et al.*, 1994 ▸; Kokoulin *et al.*, 2014 ▸; Bramhachari & Dubey, 2006 ▸; Drouillard *et al.*, 2018 ▸; Hassler *et al.*, 2011 ▸; Nichols *et al.*, 2005 ▸). Compared with their terrestrial counterparts, EPS released by marine bacteria typically contain more uronic acids (for example d-glucuronic and d-galacturonic acid; Chi & Fang, 2005 ▸; Delbarre-Ladrat *et al.*, 2014 ▸) that provide them a negative charge and serve important ecological functions. The polyanionic nature of bacterial EPS was shown to facilitate microbial adhesion and biofilm formation, and to mediate the binding and the fate of trace metal nutrients in marine systems, while the poor reactivity of bacterial EPS to microbial degradation is related to the chemical and structural complexity of these molecules and the diversity of bacteria that produce them (Decho & Gutierrez, 2017 ▸).

Carin-1, a short-tailed virus (podovirus) that infects the marine bacterium *Cobetia marina*, encodes a large protein, Dpo31, that at the sequence level shows very little similarity to any other characterized protein in public databases. We show here that Dpo31 displays EPS-lytic activity and a three-dimensional structural organization resembling that of typical tail-spike proteins. The overall structure of the bacteriophage Carin-1 was previously solved by cryo-electron microscopy (cryo-EM), highlighting the position of Dpo31 on the tail fibers with the depolymerase activity located at the distal end of the tail structure (d’Acapito *et al.*, 2023 ▸). The crystal structure revealed a trimeric, multi-modular composition with a straight, elongated arrangement. In contrast, small-angle scattering analyses of Dpo31 in solution supported kinked or bent envelopes, which are more consistent with the configuration of the protein on the virion structure solved by cryo-EM. To identify distinct functions of the individual domains, particularly those responsible for hydrolytic activity, we generated a series of constructs that either isolated individual domains or lacked specific domains. Heterologous expression of truncated variants of Dpo31 revealed that the enzymatic activity is carried by the central module designated domain 3 (D3).

## 2. Methods

### 2.1. Bacterial and viral strains

*Cobetia marina* (DSMZ 4741) is naturally infected by Carin-1, a short-tailed virus (podovirus) isolated from the surface water of the bay of Brest at the long-term monitoring station SOMLIT (4° 33′ 07.19 W, 48° 21′ 32.13 N; Lelchat *et al.*, 2019 ▸). Infected *C. marina* was grown from cryogenized stocks in Marine Broth 2216 (DIFCO), from which Carin-1 was purified. A stock suspension of purified Carin-1 bacteriophages was stored at 4°C in autoclaved SM buffer (5.8 g l^−1^ NaCl, 2 g l^−1^ MgSO_4_, 50 ml 1 *M* Tris–HCl pH 7.5 in Milli-Q water) as described in Lelchat *et al.* (2019 ▸).

### 2.2. Bacterial EPS production

Cryogenized stocks of *C. marina* were streaked onto Marine Agar (DIFCO) and incubated overnight at 25°C. A single colony was then pre-cultivated overnight at 25°C and 200 rev min^−1^ in liquid Marine Broth enriched with 30 g l^−1^ glucose to induce EPS production. Once the exponential phase was reached, the pre-culture was inoculated [1%(*v*/*v*)] in 500 ml glucose-enriched Marine Broth and incubated for 72 h (25°C, 150 rev min^−1^).

To purify the produced EPS, cultures were clarified at 7000*g* for 20 min (4°C). Supernatants were collected, filtered on 0.2 µm Stericup filtration units (PES membrane, GP Millipore) and concentrated to approximately 50 ml via tangential flow filtration using a 30 kDa cutoff Vivaflow 200 cassette (PES membrane, Sartorius). The concentrate was dialyzed against Milli-Q water (5 × 500 ml) in order to desalt the medium. Finally, the solution was frozen at −80°C and lyophilized.

### 2.3. Heterologous expression, production and purification of Dpo31

The wild-type Dpo31 (or, in brief, Dpo31) coding sequence (Supplementary Fig. S1) was cloned using a pFO4 plasmid [vector modified from pET-15b (Novagen, USA) to be compatible with the BamHI/EcoRI ligation strategy (Groisillier *et al.*, 2010 ▸)]. This plasmid generates a hexahistidine tag at the N-terminus of the recombinant protein and contains an ampicillin-resistance gene and a LacI gene, as well as the T7 promoter. Expression vectors (pFO4) were digested either by BamHI/EcoRI or XhoI/NsiI. For each targeted sequence, restriction sites recognized by BamHI, EcoRI or their isocaudomers (BglII and MfeI, respectively) were sought using the *Geneious* software (or XhoI and NsiI/SalI and PstI). The standard scheme for primer design was defined for the forward primers as 5′-[hexa-A tail]-[BamHI or BglII]-[start codon ATG if necessary]-[hybridization site]-3′ and for the reverse primers as 5′-[hexa-A tail]-[EcoRI or MfeI]-[stop anticodon]-[hybridization site]-3′. Oligonucleotides for PCR were purchased from Operon Biotechnologies GmbH, Cologne, Germany. PCR amplification was performed on a GeneAmpR PCR System 2700 (Applied Biosystems, USA). The thermocycle utilized was denaturation at 95°C for 5 min and 30 cycles of denaturing at 95°C for 30 s, annealing at 50°C for 30 s and polymerization at 72°C for 4 min. *Escherichia coli* strain DH5α was used for standard cloning procedures and *E. coli* strain BL21 (DE3) was used for gene-expression experiments. Both were grown in Luria–Bertani (LB) liquid medium or on LB solid medium, as described by Sambrook & Gething (1989 ▸). For expression tests in 3 ml cultures, an auto-inducible ZYP5052 medium was used (Studier, 2005 ▸). All media were supplemented, when necessary, with 100 µg ml^−1^ ampicillin (sodium salt).

The *E. coli* BL21 (DE3) clones expressing soluble Dpo31 were grown at 37°C overnight in LB medium containing 100 µg ml^−1^ ampicillin. The culture was then diluted 1:100 with auto-inducible ZYP5052 medium (Studier, 2005 ▸; Groisillier *et al.*, 2010 ▸) containing 100 µg ml^−1^ ampicillin in a final volume of 500 ml and subjected to further incubation at 20°C for 72 h (baffled flasks, 300 rev min^−1^). Cells were recovered by centrifugation (35 min, 4°C, 3000*g*), resuspended in 20 ml buffer *A* (50 m*M* Tris–HCl pH 8.0, 500 m*M* NaCl, 20 m*M* imidazole) and chemically lysed as described previously (Groisillier *et al.*, 2010 ▸). Afterwards, the lysate was clarified at 12 000*g* for 30 min at 4°C and the supernatant was filtered at 0.22 µm. The supernatant was loaded onto an IMAC column charged with NiCl_2_ (0.1 *M*) and pre-equilibrated with buffer *A*. The column was washed with buffer *A* and the protein was eluted with a linear imidazole gradient produced by mixing buffer *A* and buffer *B* (50 m*M* Tris–HCl pH 8.0, 500 m*M* NaCl, 500 m*M* imidazole) at a flow rate of 1 ml min^−1^. Elution started with an imidazole concentration around 175 m*M* (35% buffer *B*) for Dpo31. The different fractions were analyzed by SDS–PAGE and fractions of interest were concentrated on Amicon Ultra-15 (10 kDa, Merck Millipore) to reach a volume of 2 ml. The protein was then injected onto a Sephacryl S-200 size-exclusion column (GE Healthcare) pre-equilibrated with buffer *C* (50 m*M* Tris–HCl pH 8.0, 300 m*M* NaCl). Absorbance was followed at 280 nm and fractions containing the enzyme were collected and pooled. A concentration step was added using an Amicon Ultra-15 until a volume of 1–2 ml was reached. This two-step purification allowed the final production of 47.3 mg of the recombinant Dpo31 protein starting from 500 ml culture. The molecular mass observed on SDS–PAGE was around 85–90 kDa for Dpo31, which is consistent with the theoretical size of the protein (86 795.47 Da; Fig. 4).

Cultures involving selenomethionine (SeMet) were performed similarly except that the auto-inducible ZYP5052 medium was replaced by PASM-5052 (Guerrero *et al.*, 2001 ▸). Despite the identical protocol, production and purification of the protein labeled with SeMet in 500 ml culture yielded 16.4 mg. The protein reacted differently on the IMAC column and eluted at an imidazole concentration of 80 m*M* (16% buffer *B*). The monodispersity of the protein samples was verified by DLS measurements (data not shown).

### 2.4. Heterologous expression, production and purification of Dpo31 domains Dpo31-ΔD1, D3* and D4

The domain boundaries of individual domains and constructs (Supplementary Fig. S1) were defined by the crystal structure of Dpo31. Constructs containing sequences corresponding to the designed variants were synthesized by GenScript. In brief, the open reading frames were amplified by PCR using primers that incorporated specific restriction sites compatible with our ligation strategies. The amplification products were cloned into the pET-15b vector, which encodes an N-terminal His_6_-tag. Recombinant plasmids were used to transform *E. coli* strain DH5α. The validated plasmids were then used to transform *E. coli* strain BL21(DE3). The transformed BL21(DE3) strains were grown overnight at 37°C in LB medium supplemented with ampicillin. After the initial small-scale production, only the individual domains D1, D2, D3*, D4 and Dpo31-ΔD1 indicated soluble expression (Supplementary Fig. S2) and were produced in larger cultures. The cultures were diluted 1:100 in 500 ml auto-inducible ZYP5052 medium (4 l to produce 3.2 mg of D3*) containing ampicillin and incubated at 20°C until saturation. After centrifugation at 5000*g* for 10 min at 4°C, the pelleted bacteria were stored at −20°C. The cells were resuspended in buffer consisting of 50 m*M* Tris–HCl pH 8.0, 100 m*M* NaCl, supplemented with 15 m*M* imidazole, a protease-inhibitor cocktail and DNase. The bacteria were disrupted using a French press, followed by centrifugation at 12 000*g* for 90 min at 4°C.

The supernatant was applied onto a HisTrap column (GE Healthcare) with Ni–NTA resin or TALON cobalt resin in the case of Dpo31 D3*. After washing, the bound proteins were eluted with a linear gradient of imidazole ranging from 15 m*M* to 1 *M*. The eluted proteins were collected and concentrated using Amicon Ultra centrifugal filters (Merck Millipore). The subdomains D1 and D2 rapidly aggregated and did not reach reasonable concentrations. The concentrated samples were subsequently purified on a Superdex 200 size-exclusion chromatography column (or Superdex 75 for Dpo31 D3*; GE Healthcare) using a buffer consisting of 50 m*M* Tris–HCl pH 8.0, 100 m*M* NaCl (Fig. 4). Finally, the proteins were concentrated using adapted Amicon Ultra-10 (Ultra 3 for D3* and D4) until a volume of about 1 ml was reached. The final concentrations for Dpo31-ΔD1, D3* and D4 were 26.9, 5.8 and 9.9 mg ml^−1^, respectively.

### 2.5. EPS analysis by size-exclusion chromatography (SEC-RI)

Prior to the analyses, samples were thawed, diluted at a ratio of 1:2 (EPS final concentration 1 mg ml^−1^) and filtered through a 0.45 µm PES filter (Sartorius). Analyses were performed using an UltiMate 3000 HPLC system. Volumes of 50 µl were injected onto two serial Superdex 10/300 columns (GE Healthcare) at 0.5 ml min^−1^. A buffer composed of 50 m*M* Tris–HCl, 0.5 *M* NaCl and 8 m*M* MgSO_4_ pH 7.5 was used as the solvent. Signal was acquired by refractometry during 120 min after injection (Supplementary Fig. S3).

### 2.6. EPS degradation assays

EPS degradation assays were performed to characterize the mode of action and physico-chemical stability of Dpo31. All assays were conducted in triplicate using purified bacterial EPS solutions [0.2%(*w*/*v*)] incubated with purified Dpo31 at a final concentration of 100 ng ml^−1^ in SM dilution buffer (50 m*M* Tris–HCl, 0.5 *M* NaCl, 8 m*M* MgSO_4_ pH 7.5). Incubation conditions and analytical procedures were adapted according to the parameters being assessed.

To determine whether Dpo31 exhibits lyase or hydrolase activity, EPS degradation was monitored at 25°C by measuring the absorbance at 235 nm, which indicates the formation of unsaturated bonds characteristic of lyase-mediated cleavage. The absorbance at 235 nm was recorded every 5 min for 1 h and after 5 h of incubation. No increase in absorbance was detected, indicating that Dpo31 activity was due to a hydrolytic reaction (Supplementary Fig. S7).

For stability assays, EPS solutions were incubated with Dpo31 for 1 h with agitation at 200 rev min^−1^ in a final volume of 200 µl. Reactions were terminated by heat inactivation at 90°C for 5 min, and samples were stored at −80°C until analysis. Temperature tolerance was assessed over a range of 5–60°C. pH dependence was evaluated at 25°C using 100 m*M* buffer systems, including Tris–maleate (pH 5.5–8.0), Tris–HCl (pH 7.5–9.0) and glycine–NaOH (pH 8.5–10.5). Salinity tolerance was tested at 25°C in SM buffer with NaCl concentrations ranging from 0 to 750 m*M*, with or without supplementation with 8 m*M* MgSO_4_·7H_2_O (Fig. 7). Control reactions containing EPS only were processed under identical conditions to assess potential auto-hydrolysis. Low-molecular-weight degradation products were recovered after filtration over a 100 kDa Vivaspin column and were analyzed by size-exclusion chromatography to visualize degradation patterns. Relative Dpo31 activity was calculated by normalizing all values to the highest activity observed within each series, which was set to 100%.

### 2.7. EPS depolymerase activity measured by DLS and MALS

To test for depolymerase activity of the different constructs, 10 m*M* Dpo31-ΔD1, D3* or D4 protein solution was incubated with 30 mg EPS solubilized in 100 ml buffer (50 m*M* Tris–HCl pH 8, 100 m*M* NaCl) for 24 h. Dynamic light-scattering (DLS) curves (Malvern Instruments) were recorded for separated EPS and protein solutions before incubation and after incubation (Supplementary Fig. S6). Only incubation with DPO31-ΔD1 led to a significative shift in EPS scattering indicative of degradation, meaning that D1, D3* and D4 are not sufficient and necessary for depolymerase activity. The catalytic activity is thus most likely located within the D3 domain.

The size range of the polymers and their hydrolysis products was assessed by high-performance size-exclusion coupled to a multi-angle light-scattering detector (HPSEC-MALS; Supplementary Fig. S4). The chromatographic system consisted of a Dionex Ultimate 3000 pump and autosampler coupled to a refractometer (Optilab rEX, Wyatt Technology) and a multi-angle light-scattering detector (Dawn Heleos, Wyatt Technology). Three columns connected in series were used for separation: Shodex SB-805, SB-804 and SB-803-HQ (8 × 300 mm each), with a separation domain extending between 10^3^ and 4 × 10^6^ g mol^−1^. A 0.1 *M* lithium nitrate solution containing 0.03% sodium azide, filtered through a 0.1 µm Durapore membrane (PES, Millipore), was used as the eluent. Elution was performed at 0.5 ml min^−1^ and at room temperature (21 ± 2°C). The data were recorded and analyzed using the *Astra* 5 software (Wyatt Technology).

### 2.8. Crystallization and structure resolution of Dpo31

Crystallization experiments for the His-tagged Dpo31 (828 residues) from phage Carin-1 were set up in a first step by screening 3 × 96 conditions using the JCSG+, PEGs Suite and PACT (Qiagen) commercial kits with a nano-drop dispensing robot (Gryphon). The screening solutions were dispensed into Greiner 96-well trays (80 µl) with sitting drops composed of 0.2 µl protein solution and 0.1 µl well solution. The best hit was further optimized by the hanging-drop vapor-diffusion method at room temperature by mixing 2 µl protein solution (12 mg ml^−1^) with 1 µl reservoir solution consisting of 1.2 *M* ammonium sulfate, 100 m*M* citric acid pH 5.0. Crystals of SeMet-labeled Dpo31 were obtained by the same method, mixing 2 µl protein solution (14.7 mg ml^−1^) with 1 µl reservoir solution consisting of 1.6 *M* ammonium sulfate, 100 m*M* citric acid pH 4.6 or 4.7. All crystals were flash-cooled in liquid nitrogen in reservoir solution containing between 15 and 25%(*v*/*v*) glycerol.

X-ray diffraction data were collected on the PROXIMA-1 beamline at the SOLEIL synchrotron, St Aubin, France. Data processing was performed using the *XDS* package (Kabsch, 2010 ▸). Since the diffraction data for Dpo31 were highly anisotropic, elliptical cutoffs were applied using the *STARANISO* program (Tickle *et al.*, 2016 ▸) to final resolutions of 2.2, 2.9 and 2.15 Å for Dpo31, SeMet Dpo31 and Dpo31-ΔD1, respectively (Table 1 ▸). Experimental phases were initially obtained from a selenium-derivatized (SeMet) crystal. A single-wavelength anomalous diffraction (SAD) data set was collected at the Se edge (λ = 0.979 Å). The selenium sites were identified using *HKL*2*MAP* (Pape & Schneider, 2004 ▸) and experimental phases were calculated with *Phaser* (McCoy *et al.*, 2007 ▸) and refined by density modification using *Parrot* (Cowtan, 2010 ▸).

The initial maps were not readily interpretable, but the presence of a left-handed parallel β-helix domain was clearly distinguishable. A search model (a 130-amino-acid fragment of PDB entry 3suc) identified using *HHpred* (Zimmermann *et al.*, 2018 ▸) could be positioned in the electron density using phased rotation and translation functions as implemented in *MOLREP* (Vagin & Teplyakov, 2010 ▸). Subsequently, several rounds of phase improvements were conducted by combining experimental and model phases, again using *Phaser* (McCoy *et al.*, 2007 ▸), followed by density modification with *Parrot* (Cowtan, 2010 ▸). The resulting model underwent iterative cycles of manual reconstruction in *Coot* (Emsley *et al.*, 2010 ▸) and refinement in *BUSTER* (Bricogne *et al.*, 2022 ▸). Phase extension was subsequently carried out using the final model for molecular replacement against a higher resolution native dataset. The final models were evaluated using *MolProbity* (Chen *et al.*, 2010 ▸).

### 2.9. Crystallization and structure determination of Dpo31 constructs

The protein constructs Dpo31-ΔD1 (26.9 mg ml^−1^), D3* (5.8 mg ml^−1^) and D4 (9.9 mg ml^−1^) were similarly screened against 3 × 96 conditions using the JCSG+, PEG Suite and PACT commercial kits. However, none of the trials for D3* led to exploitable conditions, even after additional screening with the SaltRX and PEGs II kits. The best crystals of Dpo31-ΔD1 (residues 103–828) were obtained by mixing 2 µl protein solution (26.9 mg ml^−1^) with 1 µl reservoir solution consisting of 20% PEG 3350, 200 m*M* potassium nitrate. The best crystals of D4 (residues 664–828) were obtained by mixing 2 µl protein solution (9.86 mg ml^−1^) with 1 µl reservoir solution consisting of 27%(*w*/*v*) PEG 3350, 21%(*v*/*v*) 2-propanol, 100 m*M* Tris–HCl pH 8.5. Crystals of Dpo31-ΔD1 and D4 were flash-cooled in liquid nitrogen in reservoir solution containing 10 and 12%(*v*/*v*) glycerol, respectively.

X-ray diffraction data were collected on the PROXIMA-2 beamline at the SOLEIL synchrotron, St Aubin, France. Data processing was performed using the *XDS* package (Kabsch, 2010 ▸). The structure of Dpo31-ΔD1 was phased by molecular replacement with *MOLREP* (Vagin & Teplyakov, 2010 ▸) using the native Dpo31 structure after deleting the D1 domain, and was reconstituted as a trimer by applying the ‘Generate symmetry mates’ option in *Coot* (Emsley *et al.*, 2010 ▸). Iterative cycles of structure refinement under strong NCS restraints and manual reconstruction were carried out with *BUSTER* (Bricogne *et al.*, 2022 ▸) and *Coot* (Emsley *et al.*, 2010 ▸), respectively. The structure of D4 was phased by molecular replacement using *MOLREP* (Vagin & Teplyakov, 2010 ▸) and the coordinates of the D4 domain of Dpo31, and was refined with *BUSTER* (Bricogne *et al.*, 2022 ▸). Final refinement statistics for both crystal structures are given in Table 1 ▸.

### 2.10. Small-angle X-ray scattering (SAXS) analyses

SAXS measurements were conducted on the SWING beamline at the SOLEIL synchrotron, St Aubin, France and were performed at 15°C. The SAXS data were measured using the same buffer as for crystallization trials (50 m*M* Tris–HCl pH 8, 100 m*M* NaCl). The scattering vector is defined as *q* = 4πsinθ/λ, where 2θ is the scattering angle. Data were collected covering a *q* range of 0.005–0.5 Å^−1^. Data were recorded using an AVIEX170170 CCD detector at a distance of 1.807 m (λ = 1.033 Å). Protein solutions of Dpo31, Dpo31-ΔD1, D3* and D4 were prepared at the final concentrations given in Table 2 ▸. SAXS data were collected on samples eluting from an online size-exclusion high-performance liquid chromatography (SEHPLCBio-SEC3Agilent) column directly connected to the SAXS measuring cell. Volumes of protein sample ranging from 60 to 120 µl were injected and eluted directly into the SAXS capillary at a flow rate of 0.2 ml min^−1^. The elution buffer consisted of 50 m*M* Tris–HCl pH 8.0, 100 m*M* NaCl, identical to those used for the size-exclusion chromatography purification step. 250 SAXS frames were collected continuously during elution with a frame duration of 1.5 s and a dead time between frames of 0.5 s. 100 frames accounting for buffer scattering were collected before the void volume. SAXS data were normalized to the intensity of the incident beam, and background (*i.e.* the elution buffer) was subtracted using the program *FoxTrot* (David & Pérez, 2009 ▸). The experimental curves were analyzed using *PRIMUS* as part of the *ATSAS* suite (Franke *et al.*, 2017 ▸) to calculate the *R*_g_ value in the Guinier region, and *GNOM* (Svergun, 1992 ▸) was used to calculate the *P*(*r*) and *D*_max_ values. The experimental curve was compared with the theoretical diffusion curves for the crystal structure and the cryo-EM structure using *CRYSOL* (Franke *et al.*, 2017 ▸) as implemented in the *ATSAS* suite. *GASBOR* (Svergun *et al.*, 2001 ▸) or *DAMMIF* (Franke & Svergun, 2009 ▸) were used in subsequent steps for data interpretation and *ab initio* modeling to find the best shapes that fit the experimental data.

## 3. Results

### 3.1. Crystal structure determination of the viral enzyme Dpo31

**Figure 1:**
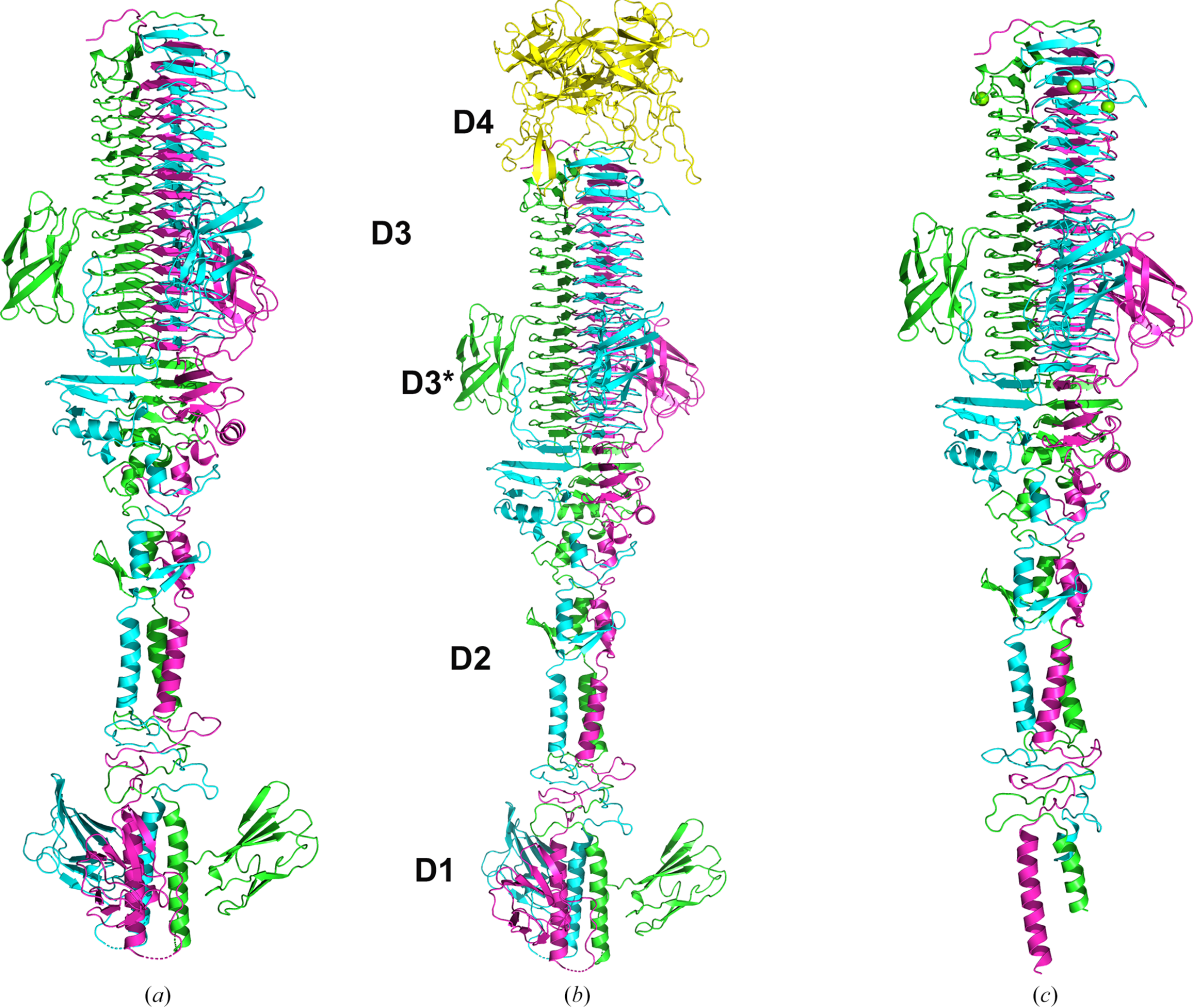
Ribbon representation of the trimeric organization in the crystal structures of (*a*) native Dpo31, (*b*) Dpo31 and the docked most probable position of the trimeric D4, which although present in the construction was not visible in the electronic density of the crystal structure, and (*c*) of Dpo31-ΔD1. The three individual protein chains are colored green, cyan and magenta, respectively. The docked ribbon representation of the D4 trimer, as defined in the crystal structure, is colored yellow. The N-terminal end of the protein is located at the bottom and the C-terminus is positioned at the top of the representations in (*a*), (*b*) and (*c*). The protein attaches to the phage tail through the N-terminal D1 domain.

After having identified the polysaccharide depolymerase of Carin-1 within the phage genome (Mocaër, 2019 ▸), the targeted gene sequence was cloned and heterologously expressed in *E. coli*. EPS-degradation assays were successful with the recombinant protein, confirming that the heterologous protein was produced in an active form. Larger quantities (almost 50 mg) were then produced and purified to homogeneity in order to structurally and biochemically characterize the enzyme. Since the sequence homology to any characterized protein was very low (below 10%), and at the time of the experiments *AlphaFold* did not yet exist, SeMet-labeled Dpo31 was also successfully produced and purified. Both the native and SeMet-labeled proteins were successfully and reproducibly crystallized, and the crystals belonged to space group *H*32 with unit-cell parameters *a* = 88.2, *c* = 643.34 Å and one Dpo31 or SeMet-labeled molecule in the asymmetric unit (Table 1 ▸). The trimeric structural organization of Dpo31 was formed by three intertwined protein chains centered on the crystallographic threefold axis (Fig. 1 ▸).

**Figure 2:**
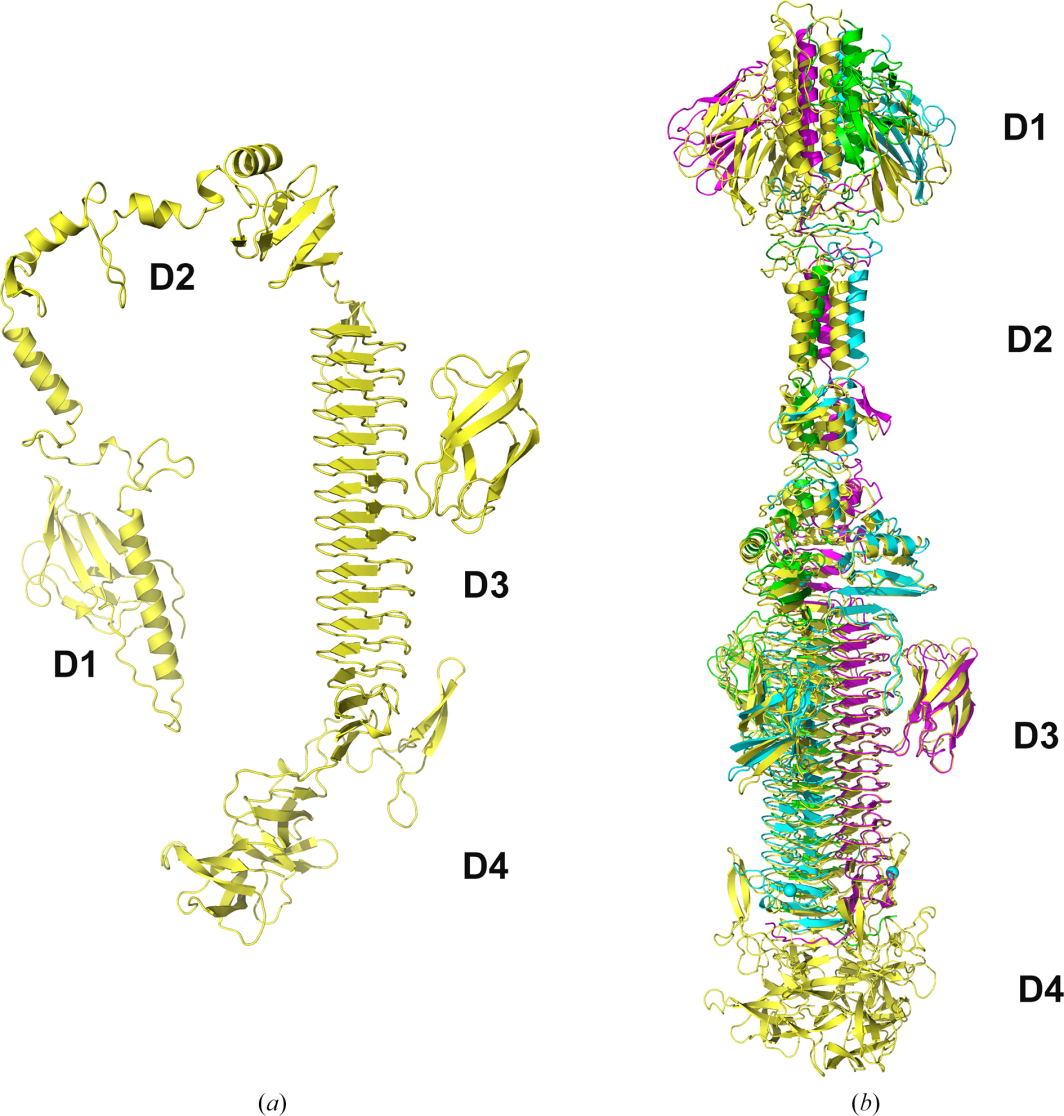
Ribbon representation of *AlphaFold*-predicted models of Dpo31. The different domains from D1 to D4 are labeled. (*a*) Prediction of full-length Dpo31 when submitting the sequence as a monomer, without specifying the oligomeric state, resulting in a curved overall shape. (*b*) Superimposition of the *AlphaFold*-predicted model (yellow), when specifying the oligomeric state as a trimer, onto the crystal structure of Dpo31 (the three monomers are colored magenta, cyan and green, respectively). D4 is absent in the crystal structure and D3 of both models fits well, but the presence of a clear twist or rotation, starting from D2 and increasing towards D1, results in a large discrepancy between these domains in the predicted and experimental structures.

When submitting the sequence to *AlphaFold*3 without knowing the oligomeric state, the overall secondary structures of the different subdomains are well predicted, but the relative positions of these are incorrect; the structure is curved, forming almost a U shape (Fig. 2 ▸*a*), and far from the bent form observed by SAXS or cryo-EM. In contrast, when submitting the sequence and asking for a trimer, *AlphaFold*3 predicts a straight form, similar to that observed in the crystal structure, but the root-mean-square deviation (r.m.s.d.) between the predicted and the experimental structure is 4.02 Å for 395 C^α^ atoms out of 652, which hampers solution of the structure by molecular replacement using this structure as a model. Indeed, while D3 is well superimposed, the *AlphaFold*3 model is twisted in domains D2 and D1 (Fig. 2 ▸*b*) with respect to the crystal structure, and the change in arrangement would have been difficult to detect when preparing a suitable model for molecular replacement.

**Figure 3:**
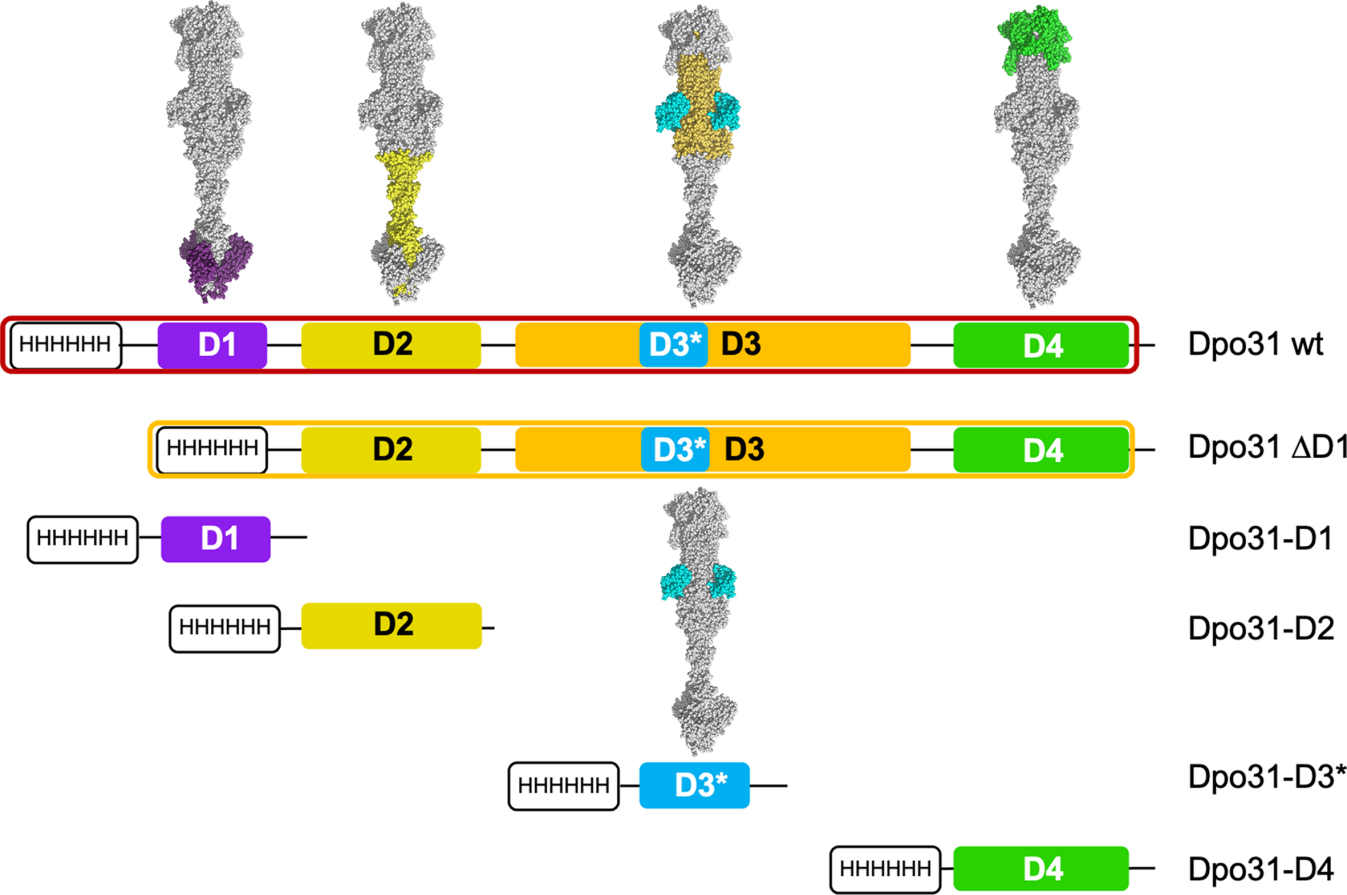
Schematic representation of the domain structure of Dpo31. Colored boxes in the linear diagram denote individual domains: D1 (purple), D2 (yellow), D3 (orange), D3* (cyan) and D4 (green). Above the diagram, representative surface renderings of the assembled particle are shown with the corresponding domain densities colored as in the schematic. In all cases, the mature form does not include domain D4.

The 3D crystal structure of Dpo31 was solved using the SAD method at the wavelength of the Se absorption edge (λ = 0.979 Å) on a SeMet-labeled crystal at low resolution (for details, see Section 2). This preliminary model was subsequently refined against very anisotropic native data obtained from a Dpo31 crystal that diffracted to a final resolution of 2.2 Å (Table 1 ▸). The overall trimeric protein structure displayed an elongated shape 240.4 Å in length and roughly 80 Å in diameter. The crystal structure also revealed an organization into several domains, which were named D1–D4 (Fig. 3 ▸ and Supplementary Fig. S1) in analogy to other tail-spike proteins (Xiang *et al.*, 2009 ▸).

The final crystal structure could be traced from residues 8 to 669, covering D1–D3 (including D3*), while no density was identified for D4. Moreover, the crystal packing is such that there is no space for D4, and we assume that only the mature form of Dpo31, without D4, is present in the crystals. This is supported by the systematic presence of a band at 18 kDa, corresponding to the molecular weight of D4, in the SDS–PAGE of several Dpo31 constructs (Supplementary Fig. S2).

The N-terminal domain D1 has a classical β-sandwich fold composed of residues 8–87 and is connected, through about 23 residues with no defined secondary structure, to the first helix of a coiled-coil region of domain D2. Domain D2 is composed of two three-helix-coil regions separated by a loop junction, and is overall composed of residues 102–256. The largest domain, D3 (including D3*), comprises residues 257–663 and is composed of a trimeric left-handed parallel β-helix that forms a ladder of 17 parallel β-strands. These β-helices are tightly packed against the equivalent β-helices of the two other monomers, together forming the core of the structure, which is typical of viral tail-spike proteins (d’Acapito *et al.*, 2023 ▸; Smith *et al.*, 2005 ▸; Figs. 1 ▸*a* and 1 ▸*b*). An additional domain that we labeled D3* protrudes from the central core structure, inserted from residues 434 to 536, and displays an immuno­globulin (Ig-like) fold.

**Figure 4:**
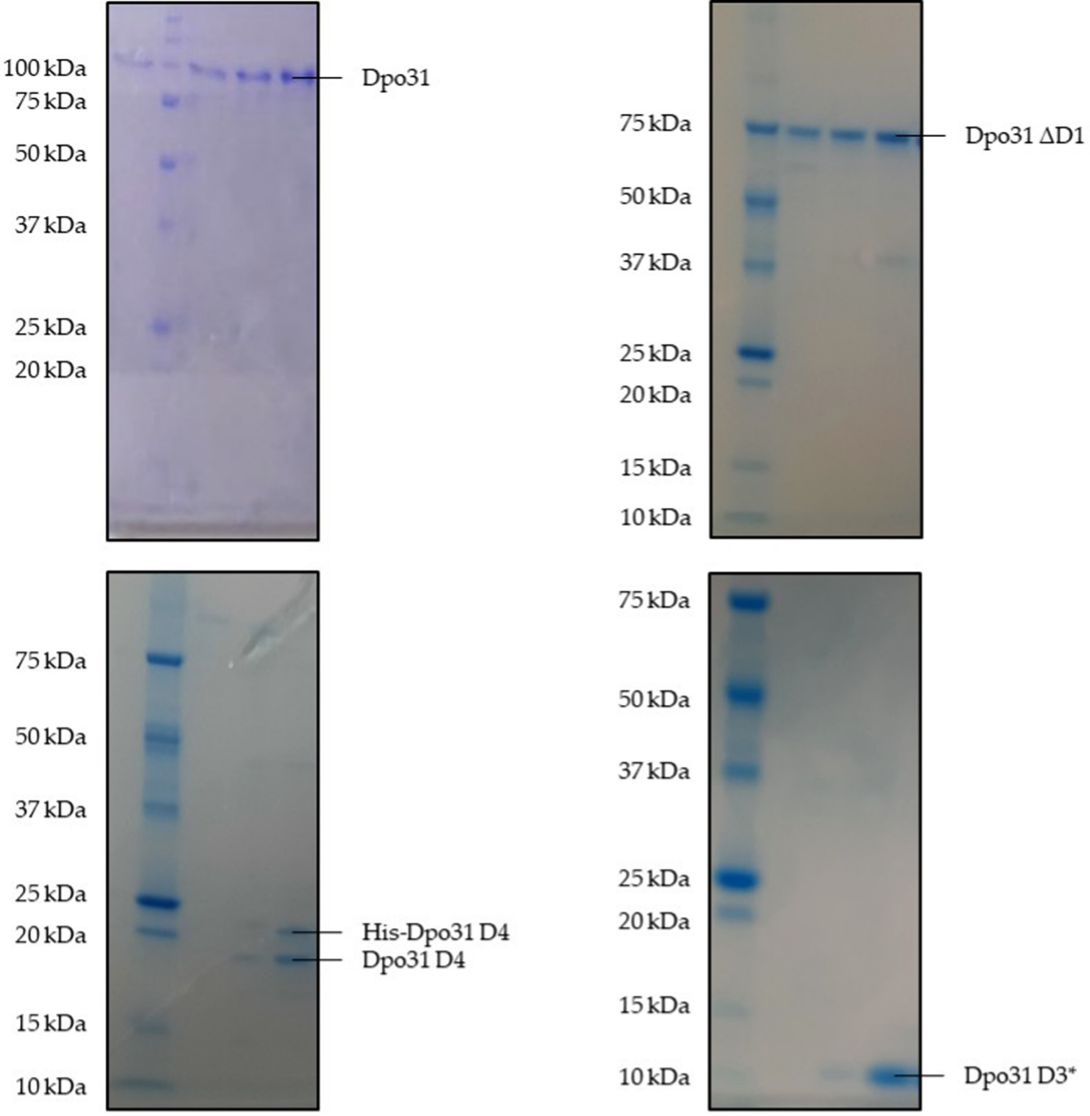
SDS–PAGE analysis of purified Dpo31 constructs. A single band is observed for each construct: full-length Dpo31 (88.7 kDa), Dpo31-ΔD1 (77.7 kDa) and Dpo31-D3* (10.5 kDa), except for Dpo31-D4, for which two bands, corresponding to the tagged (19.5 kDa) and cleaved (17.5 kDa) forms, are visible.

When discovering the complex domain organization of this large tail-spike protein, we decided to produce truncated forms and individual domains, with the aim of better localizing the catalytic activity but also to understand the importance of subdomains for the overall structural organization. Not knowing whether some of the domains work together or whether individual domains could fold correctly when isolated, we tested each domain alone, as well as omitting each domain, and several combinations two by two (Supplementary Fig. S1). While 11 different constructs were cloned (see the scheme in Supplementary Fig. S1), only Dpo31-ΔD1 and the individual domains D1, D2, D3* and D4 were expressed solubly (Fig. 4 ▸ and Supplementary Fig. S2). Dpo31-ΔD1, D3* and D4 were purified in sufficient quality and quantity to set up crystallization trials and undertake SAXS measurements. The constructs D1 and D2 aggregated rapidly and appeared not to be suitable for further biochemical characterization.

### 3.2. Crystal structure determination of the truncated constructs Dpo31-ΔD1 and D4

We designed and expressed a series of constructs covering the four domains of the tail spike (D1–D4) to evaluate their contributions to folding and solubility. The intact construct covering domains D1–D4 and the fragment D2–D3–D4 (Dpo31-ΔD1) were soluble and purified readily (yields of ∼50.0 mg and 34.1 mg per 500 ml culture, respectively; Fig. 4 ▸), whereas most fragments lacking D4 (D1–D2–D3, D2–D3, D3, D1–D2) were insoluble (Supplementary Fig. S2). The isolated D4 domain is intrinsically soluble but is expressed at low yield (∼5.9 mg in 500 ml culture). D2 alone shows incomplete solubility and a tendency to aggregate, and D1 is poorly soluble. These results are consistent with a structural model in which D2 forms the trimeric core required for assembly and D4 functions as an auto-chaperone: D4 facilitates final folding only in the context of a properly formed D2 scaffold (for example D3–D4 is insoluble, while D2–D3–D4 is soluble). The observations also suggest that domain boundaries and/or context-dependent cleavage of D4 can influence fragment solubility. Because solubility and proper assembly depended critically on the D2 trimeric scaffold and the presence of D4, we focused crystallographic efforts on truncated variants that retain the architecture. Crystals were successfully obtained for Dpo31-ΔD1 and D4, while D3* did not crystallize within roughly 500 crystallization trials. The crystals of Dpo31-ΔD1 belonged to space group *P*2_1_, with unit-cell parameters *a* = 93.7, *b* = 75.6, *c* = 124.9 Å, β = 103.3° and three molecules in the asymmetric unit (Table 1 ▸), corresponding to the trimeric structure of the protein (Fig. 1 ▸*c*). Domain D2 was more disordered in this truncated construct; nevertheless, the monomeric chains could be traced from residues 112, 103 and 109 for the three monomeric chains, respectively. All three chains ended at residue 663, which is slightly shorter than for the native Dpo31 protein. Chain-by-chain superimposition between the native monomer and each of the Dpo31-ΔD1 monomers leads to root-mean-square deviations (r.m.s.d.s) of 1.94 Å (*A*–*A*), 1.04 Å (*A*–*B*) and 1.94 Å (*A*–*C*), respectively. Overall, no major conformational changes are observed, but the helices of domain D2 display much higher *B* factors, more than twice as high in the truncated protein with respect to the native Dpo31 protein.

**Figure 5:**
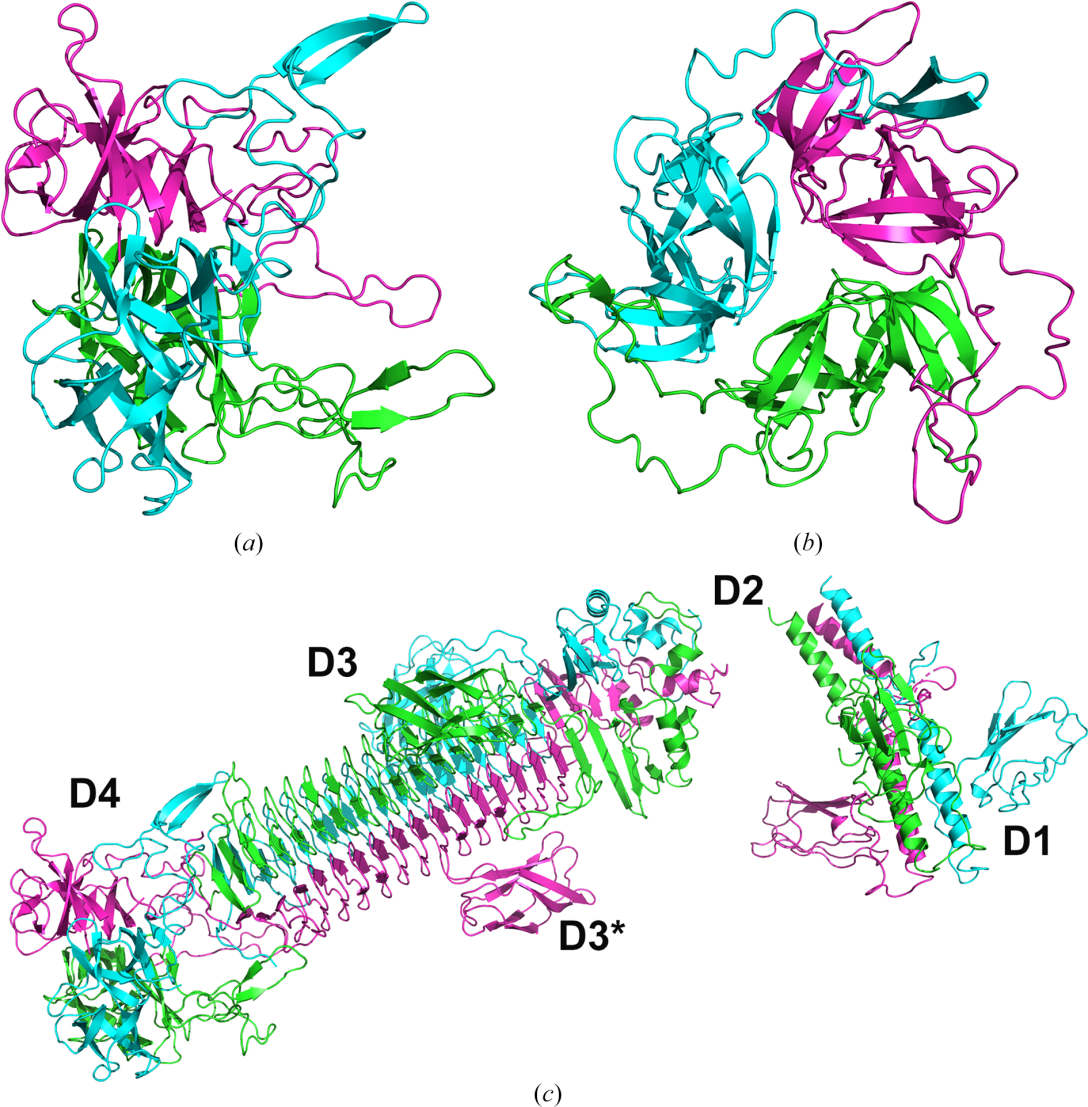
Ribbon representation of the structural organization of D4 as observed in the crystal structure of the isolated domain. (*a*) Side view and (*b*) top view of the trimeric organization of D4. (*c*) Ribbon representation of the bent structure of Dpo31 that was fitted into the cryo-EM electron density (d’Acapito *et al.*, 2023 ▸). Three disordered regions between D1 and D2, within D2 and between D2 and D3 are responsible for bending.

The crystals of D4 belonged to space group *P*3_2_21, with unit-cell parameters *a* = *b* = 69.7, *c* = 533.4 Å, and contained three independent copies (unrelated by any rotational symmetry) of the trimeric organization of the monomeric D4 domains, leading to a total of nine molecules of D4 in the asymmetric unit. The three, biologically relevant, D4 trimers superimpose well (the r.m.s.d.s of superimpositions between trimers range between 1.27 and 1.38 Å) and their presence confirms that this domain is able to form the trimeric organization in the absence of the rest of the protein (Figs. 5 ▸*a* and 5 ▸*b*). All residues from Asp664 to Lys828 could be traced in the electron density, although several regions were disordered. A *DALI* search revealed that D4 matches best (*Z*-score 21, r.m.s.d. of 1.7 Å) with an equivalent domain of the tail-spike (TS) protein gp12 from the bacteriophage φ29-infecting *Bacillus subtilis* (Xiang *et al.*, 2009 ▸) with the same trimeric organization, although neither D2 or D3 of the respective TS proteins have the same level of similarity.

### 3.3. Small-angle X-ray scattering (SAXS) analyses of Dpo31 and truncated constructs

**Figure 6:**
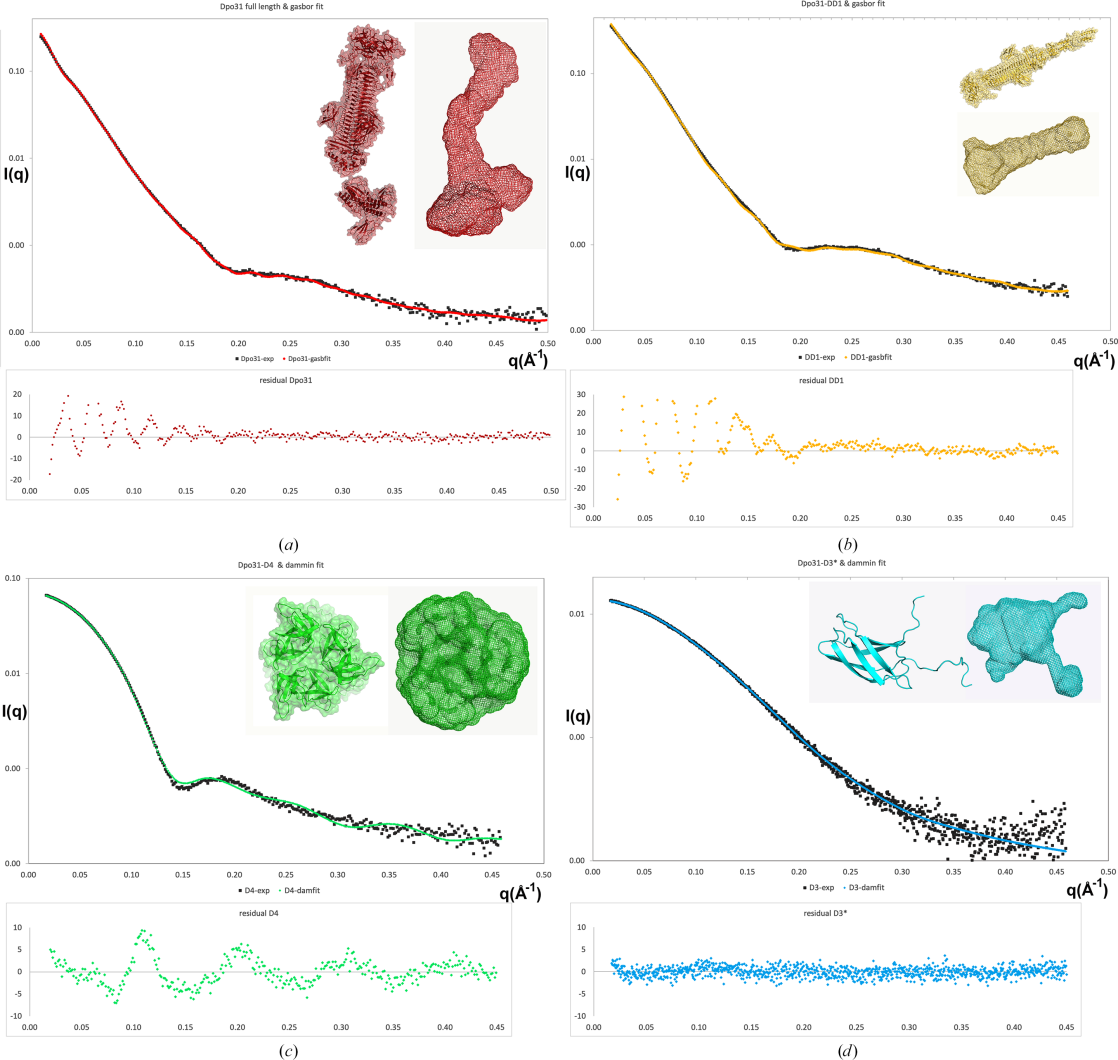
Small-angle X-ray scattering curves of Dpo31 (*a*), Dpo31-ΔD1 (*b*), D4 (*c*) and D3* (*d*). In all panels, experimental data are represented as black dots and the calculated fit by *GASBOR* (*a*) or *DAMMIF* (*b*, *c*, *d*) is colored red (native Dpo31), yellow (Dpo31-ΔD1), green (D4) and cyan (D3*), respectively. The residual of each fit is given as Δ*I*(*q*)/σ*I*(*q*) below each corresponding curve (same colors). In each panel, the insets show ribbon representations of structural coordinates (left or upper inset) and the most representative envelopes that fit the experimental data (right or lower inset); the colors are the same as described above.

To obtain further insight into the structural domain arrangement of Dpo31 in solution, we have successfully collected small-angle X-ray scattering data for native Dpo31 and the purified and stable constructs Dpo31-ΔD1, D3* and D4 (Fig. 6 ▸ and Table 2 ▸). As expected, the scattering data for Dpo31 (Fig. 6 ▸*a*), Dpo31-ΔD1 (Fig. 6 ▸*b*) and D4 (Fig. 6 ▸*c*) show that the trimeric organization is maintained in solution while, not surprisingly, D3* appears as a monomer (Fig. 6 ▸*d*).

In the case of native Dpo31 and Dpo31-ΔD1, the scattering curves are best fitted by clusters of envelopes [calculated with *DAMMIF* (Franke & Svergun, 2009 ▸) or *GASBOR* (Svergun *et al.*, 2001 ▸)], which in general points towards the presence of conformational variability and/or flexibility. In both cases, the theoretical scattering curves calculated from the crystal structure coordinates using *CRYSOL* (as implemented in the *ATSAS* suite; Franke *et al.*, 2017 ▸) only give very poor fits, which also supports the presence of conformational variability. Most interestingly, the most representative envelopes calculated for Dpo31-ΔD1 maintain the straight, elongated arrangement, while native Dpo31 envelopes are more compatible with the bent form that was previously observed in the cryo-EM data (d’Acapito *et al.*, 2023 ▸). In contrast, the theoretical scattering of the *AlphaFold* (Jumper *et al.*, 2021 ▸) and crystal structures of the D3* domain and D4 trimer, respectively, fit the experimental curve with reasonable χ^2^ values (*CRYSOL* fit in Table 2 ▸).

### 3.4. Analyses of EPS degradation by recombinant Dpo31 with SEC-RI

EPS from *C. marina*, native and degraded, were analyzed using size-exclusion chromatography coupled with a refractometer. Data recorded with native EPS showed two distinct populations: (i) a first population with an elution time peak at 30 min with a consequent front shoulder between 26 and 28 min, and with two slight following shoulders between 32 and 35 min and between 35 and 40 min, and (ii) a large peak between 44 and 63 min with a peak at 54 min of elution (Supplementary Fig. S3*a*).

After degradation by the Dpo31 enzyme, modifications appeared in both populations. These effects were observed in a noncomplete degradation state (Supplementary Fig. S3*b*) and in a final degradation step (Supplementary Fig. S3*c*). In the first case, the highest peak of the first population at 30 min faded, but the front and the shoulder remained. The shoulder at 35–40 min increased and a peak appeared at 37 min of elution. Concerning the second population, the large peak remained, but three populations appeared at 57, 59 and 62–63 min. In the case of the final degradation, the same observations were made as for the first population. However, a unique peak appeared after the large second population at an elution time of 62–63 min. The area of this latter peak was considered to be final degradation products and was taken into account for further analysis regarding the enzyme activity at different pH, temperature and salinity conditions. Similarly, degradation, although not to completion, was observed when following the reaction by HPSEC-MALS (Supplementary Fig. S4) since an additional peak with a lower molecular weight appeared after incubation with Dpo31 (Supplementary Table S1). Altogether, these results confirm that recombinant Dpo31 is able to degrade EPS produced by *C. marina*.

### 3.5. Dpo31 activity under variable temperature, pH and salt conditions

**Figure 7:**
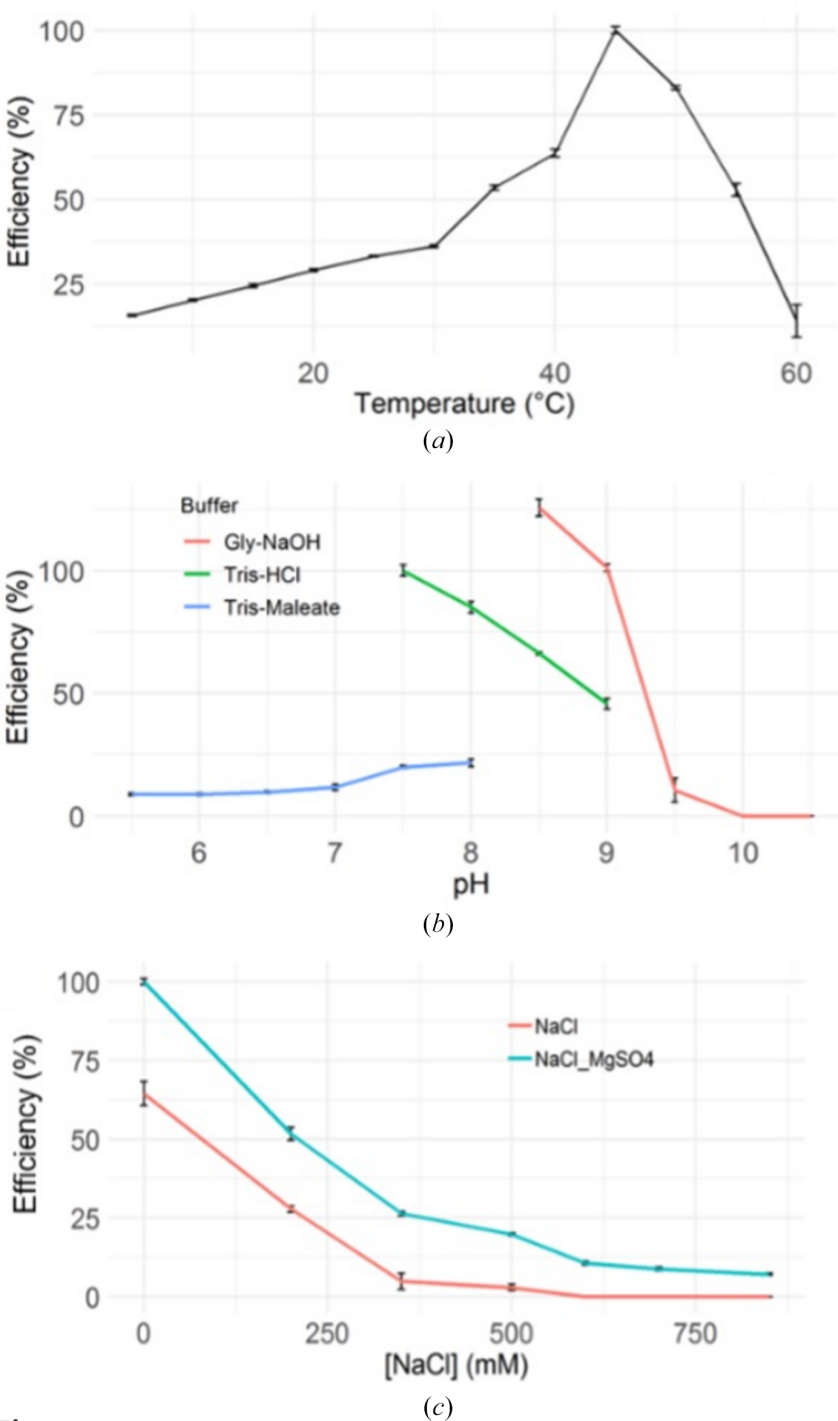
Degradation efficiencies of Dpo31 under varying conditions (*a*) temperature, (*b*) pH and (*c*) salt concentration. Relative Dpo31 activity was calculated by normalizing each series to its highest observed activity, set as 100%. For the pH series, values were specifically normalized to activity at Tris–HCl pH 7.5.

The activity of Dpo31 increased with increasing temperature from 4 to 45°C, reaching its optimal activity at 45°C (Fig. 7 ▸*a*). At this optimal temperature, Dpo31 activity was four times higher than at 13°C, which is the average seawater temperature at the Carin-1 isolation site. Above 45°C enzyme activity declined rapidly, and no activity was recorded at 60°C. This correlates well with the protein melting temperature (*T*_m_) determined by dynamic light scattering (DLS), for which we obtained a *T*_m_ of 43.72 ± 0.15°C (Supplementary Fig. S5). Regarding pH conditions, Dpo31 was functional between pH 5.5 and 10, with optimal activity at pH 8.5 in a glycine–NaOH buffer. The buffering molecule influenced the enzymatic activity, with glycine–NaOH yielding twice the activity of Tris–HCl at pH 8.5 (Fig. 7 ▸*b*). The addition of salt reduced Dpo31 activity, with the maximal activity recorded in the absence of NaCl, and a continuous decrease was observed up to a concentration of 350 m*M*. No activity was detected at NaCl concentrations above 600 m*M*. The addition of MgSO_4_ enhanced the depolymerization activity, although the same declining pattern with increasing NaCl concentration was observed (Fig. 7 ▸*c*).

### 3.6. Activity of truncated constructs as measured by dynamic light scattering

To more precisely identify the location of the enzymatic activity, EPS degradation in the presence of the various soluble and stable constructs, Dpo31-ΔD1, D3* and D4, was monitored by dynamic light scattering (DLS; Supplementary Fig. S6*a*). The size distribution of the substrate after 24 h of incubation diminished only in the presence of Dpo31-ΔD1 (Supplementary Fig. S6*b*) and not in the presence of D3* (Supplementary Fig. S6*c*) or D4 (data not shown), indicating that similar to analogous tail-spike proteins, the catalytic activity is carried by the central domain D3. While D3* alone clearly is not responsible for activity, we could not explore whether the presence of D3* is required for activity, since constructs of D3 alone were not stable in our hands.

## 4. Discussion

**Figure 8:**
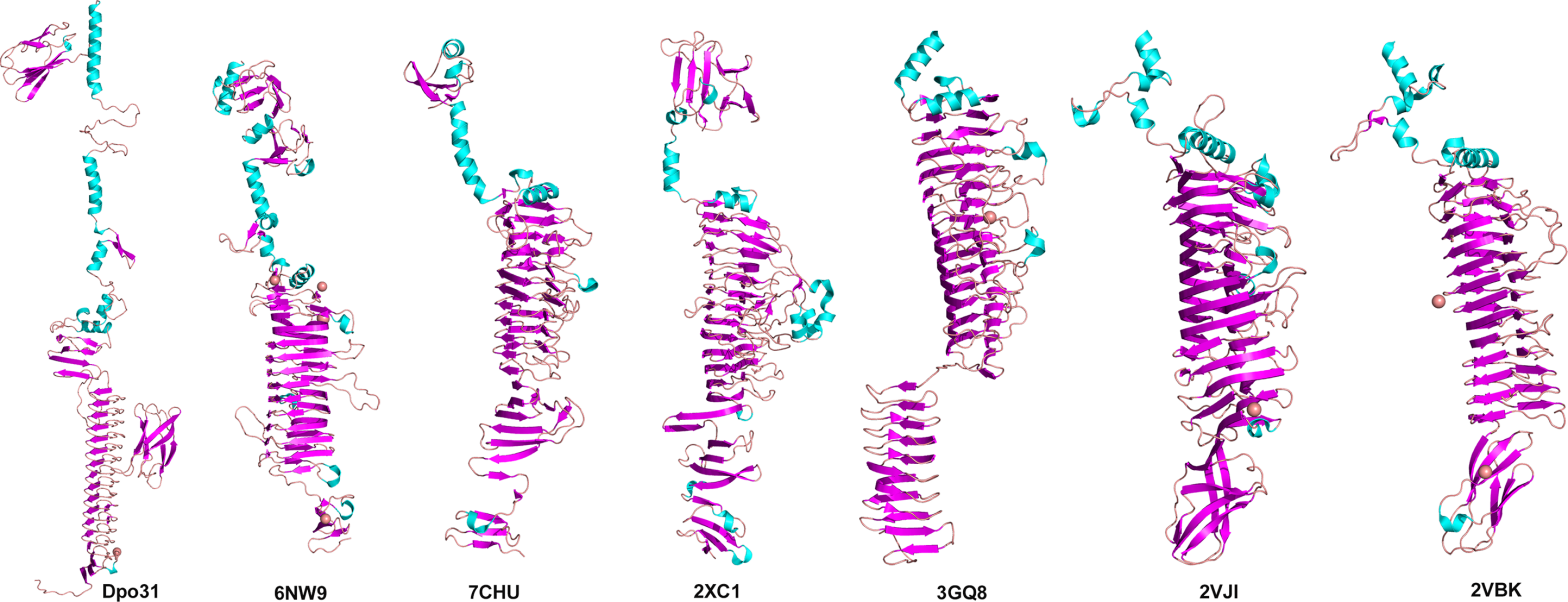
Ribbon representation comparing various tail-spike protein monomers for which crystal structures have been determined. Secondary structures are colored magenta for β-sheets, cyan for α-helices and pink for loops. The comparison highlights the large diversity of structural features leading to low sequence identities of less than 10% between proteins. The leftmost panel shows Dpo31 (our study; PDB entry 28lx) compared with CBA120-TSP3 (PDB entry 6nw9), DnGVE2 (PDB entry 7chu), P22 (PDB entry 2xc1), φ29 (PDB entry 3gq8), HK620 (PDB entry 2vji) and Sf6 (PDB entry 2vbk).

The present study, focusing on the structural and biochemical characterization of Dpo31 from the marine bacteriophage Carin-1, reveals that this protein displays a typical, although divergent, tail-spike protein fold, as illustrated in Fig. 8 ▸. Intriguingly, the crystallographic space group of Dpo31, which is *H*32, and the protein arrangement in the crystals is commonly found for tail-spike proteins (Xiang *et al.*, 2009 ▸; Smith *et al.*, 2005 ▸). Most interestingly, the distant structural similarity was not detected at the primary-sequence level, with less than 10% sequence identity of Dpo31 to any characterized protein. This genetic divergence highlights the fact that comparative genomics does not appear to be a suitable tool to assess diversity and evolutionary relationships of these viral proteins, and provides a likely explanation why these depolymerases and/or tail-spike proteins are rarely detected in marine bacteriophage genomes.

Despite a similar overall structural organization of Dpo31 compared with tail-spike proteins from various bacterio­phages (Fig. 8 ▸), such as ΔnGVE2 (Zhang *et al.*, 2020 ▸) or P22 (Seul *et al.*, 2014 ▸), it does display several distinct features providing structural novelty. In particular, while the β-helix domains D3 of ΔnGVE2 (PDB entry 7chu) and P22 (PDB entry 2xc1) have 10 and 13 helical turns, respectively, domain D3 in Dpo31 is particularly long, having 17 β-helix turns. While protruding loops or domains in the β-helix domain (D3 in our structure) have been reported, the D3* domain of Dpo31 displays an immunoglobulin-like structural arrangement that so far has not been observed in short-tail podovirus tail-spike proteins. A *DALI* search with the isolated D3* domain reveals distant similarity to sugar-binding domains (SBDs) with an Ig-like fold, such as those present in a galactan synthase (PDB entry 8d3z; *Z*-score 2.9, r.m.s.d. of 3.38 Å for D3* and SBD) and a GH2 (PDB entry 8u01; *Z*-score 2.8, r.m.s.d. of 3.89 Å for D3* and SBD). Based on these similarities in combination with our activity assays, showing that all constructs containing domain D3 degrade the EPS from *C. maritima*, we speculate that the depolymerase activity is located in the D3 domain and probably at or close to the interface with the D3* domain. Due to the low sequence similarities with other depolymerases, it is impossible at this stage to identify any residues that could play a role in the (hydro)lytic activity.

In the recent cryo-EM study of the entire Carin-1 virion by d’Acapito *et al.* (2023 ▸) it was possible to identify and position this tail-spike protein in the experimental map, allowing its relative orientation to be defined, with D1 linked to the base of the virion and D3 oriented outwards, ready to anchor to the host’s surface (Figs. 6*a* and 6*c* in d’Acapito *et al.*, 2023 ▸). Importantly, the arrangement that fitted best the cryo-EM map is bent, with three hinges located between D1 and D2, within D2 and between D2 and D3 [see Figs. 6*b* and 6*c* of d’Acapito *et al.* (2023 ▸) and Fig. 5 ▸*c*], while the overall arrangement of the Dpo31 domains in the crystal structure is perfectly aligned and straight. In contrast, our SAXS experiments showed that the arrangement observed in solution (Fig. 6 ▸*a*) is in better agreement with the bent form observed in the cryo-EM map, indicating that the straight conformation is most likely forced by crystal-packing interactions.

In the study of protein gp12 from bacteriophage φ29 by Xiang *et al.* (2009 ▸) the authors show that the C-terminal D4 domain is an ‘auto-chaperone’ aiding with the trimeric formation of the rest of the TS protein. In our study, the corresponding domain D4 was systematically absent in the expressed proteins, even if present in the cloned construction (Supplementary Figs. S1 and S2). In addition, the presence of an intense band at the size of D4 in the SDS–PAGE gels (Supplementary Fig. S2) indicates that the domain was prone to proteolytic cleavage. Together with the structural similarity that we observe, and the well conserved organization of the trimeric D4 in our study, these findings allow us to suggest that D4 plays a similar role in Dpo31. Furthermore, by comparing the ATP-binding site residues reported by Xiang *et al.* (2009 ▸) (PDB entries 3suc and 3gqk), we found that most of the key amino acids are conserved in our structure, suggesting that the overall architecture of the ATP-binding pocket is also preserved, despite the absence of ATP in our crystal structure.

Our study provides the first experimental evidence that this tail-spike protein, Dpo31, acts as a depolymerase active on the EPS secreted by *C. marina*. Moreover, we were able to locate the lytic activity in the D3 domain. Interestingly, the lytic activity does not degrade the EPS completely, in the sense that no small oligosaccharides are released as reaction products, but in contrast the depolymerase produces polysaccharide fractions with medium to high molecular weight (roughly around 225 kDa) after incubation with *C. marina* EPS. We conclude that Dpo31 seems to be very specific for a rarely occurring bond or sugar unit, which could explain the occurrence of this degradation pattern and our difficulties in characterizing the reaction products further. This highly specific cleavage of a rarely occurring bond or sugar could also explain the specificity of the depolymerase for *C. marina* EPS. Such specificity may underlie the strict host range of Carin-1 for *C. marina*.

From an ecological perspective, the depolymerization activity of Dpo31 suggests that phages may influence EPS turnover in natural marine environments in a highly selective manner. Marine bacteria are indeed known to release copious amounts of EPS into seawater, where they constitute a major component of dissolved organic matter (DOM; Decho & Gutierrez, 2017 ▸; Stoderegger & Herndl, 1998 ▸). Experiments with natural bacterial communities supplemented with EPS indicate that these macromolecules are largely resistant to microbial degradation (Stoderegger & Herndl, 1998 ▸, 1999 ▸; Zhang *et al.*, 2020 ▸), suggesting that EPS contribute to semi-labile and refractory DOM pools and long-term carbon storage. Our results demonstrate that marine phages can transform EPS through tail-bound Dpos, with efficiencies that depend on environmental parameters such as pH, temperature and salinity. Nevertheless, the physiological relevance of the observed salt sensitivity remains to be further investigated. This reveals a previously unrecognized viral mechanism capable of altering the EPS size spectrum, with potential consequences for EPS bioavailability. Considering the ubiquity of phages (Stoderegger & Herndl, 1999 ▸) and the widespread distribution of bacterial EPS (Decho & Gutierrez, 2017 ▸) in the ocean, viral Dpos may contribute to the structuring of microbial communities and the modulation of carbon fluxes, and therefore warrant further investigation.

## Supplementary Material

PDB reference: Dpo31, native, 28lx

PDB reference: ΔD1, 28mg

PDB reference: D4, 28nb

SASBDB reference: Dpo31, ΔD1, SASDYX6

SASBDB reference: D4, SASDYY6

SASBDB reference: native, SASDYZ6

Supplementary Figures and Table,. DOI: 10.1107/S2059798326005425/ni5032sup1.pdf

## Figures and Tables

**Table 1:** Data-collection and refinement parameters for crystallographic structure determination of Dpo31, Dpo31-ΔD1 and Dpo31-D4 Values in parentheses are for the highest resolution shell.

	Dpo31, SeMet†	Dpo31, native‡	Dpo31-ΔD1§	Dpo31-D4
Data collection				
Space group	*H*32	*H*32	*P*2_1_	*P*3_2_21
*a*, *b*, *c* (Å)	88.0, 88.0, 644.4	88.2, 88.2, 643.3	93.7, 75.6, 124.9	69.7, 69.7, 533.4
α, β, γ (°)	90, 90, 120	90, 90, 120	90, 103.3, 90	90, 90, 120
Resolution range (Å)	49.2–2.90 (2.98–2.90)	49.2–2.20 (2.26–2.20)	47.4–2.15 (2.21–2.15)	48.49–3.30 (3.53–3.30)
After *STARANISO*				
Measured/unique reflections	707243/16892	475253/22469	367829/59089	313214/24378
Completeness (%)	75.3 (22.2)	43.7 (5.2)	63.4 (6.7)	100 (100)
〈*I*/σ(*I*)〉	17.1 (1.1)	18.4 (2.4)	9.6 (2.3)	5.1 (1.5)
*R*_p.i.m._	0.032 (0.703)	0.026 (0.295)	0.050 (0.351)	0.137 (0.329)
*R*_merge_	0.207 (4.063)	0.115 (0.875)	0.115 (0.543)	0.526 (1.138)
Multiplicity	42.3 (37.1)	21.2 (8.9)	6.2 (3.3)	14.9 (12.9)
CC_1/2_	1.000 (0.361)	1.000 (0.787)	0.997 (0.613)	0.917 (0.568)
Refinement				
Resolution range (Å)		49.21–2.20	47.38–2.15	47.33–3.30
No. of reflections/No. in test set		22468/1092	59091/2978	24160/4233
*R*_work_/*R*_free_ (%)		25.2/27.3	21.2/24.3	24.7/25.9
No. of atoms				
Protein		4789	12050	10847
Heterogen		40	34	4
Water		43	411	90
R.m.s.d. from ideal values				
Bond lengths (Å)		0.005	0.006	0.006
Bond angles (°)		0.83	0.87	1.003
Average *B* factor (Å^2^)				
From atoms		57.3	66.7	67.5
From Wilson plot		46.2	26.8	43.6
Ramachandran plot				
Most favored (%)		95.8	97.0	83.3
Outliers (%)		0.8	0.2	3.5
*MolProbity* score		1.38	1.33	2.46
PDB code		28lx	28mg	28nb

† Diffraction data were collected from two crystals, which diffracted anisotropically to 3.33 Å along 0.894a* − 0.447b*, 3.33 Å along b* and 2.59 Å along c*.

‡ Diffraction data were collected from two crystals, which diffracted anisotropically to 3.24 Å along 0.894a* − 0.447b*, 3.25 Å along b* and 2.09 Å along c*.

§ Diffraction data were collected from one crystal, which diffracted anisotropically to 2.05 Å along 0.711a* − 0.703c*, 2.81 Å along b* and 2.49 Å along 0.42a* + 0.91c*.

**Table 2:** Experimental SAXS parameters derived from the scattering curves of the various Dpo31 constructs Theoretical molecular weights derived from the sequence are given for the monomeric forms.

Construct	Dpo31	Dpo31-ΔD1	Dpo31-D3*	Dpo31-D4
Sample concentration (mg ml^−1^)	23.6	26.9	5.8	9.86
Molecular weight of monomer (kDa)	67.5	58.2	11	18
*D*_max_ (Å)	228 ± 4	190 ± 3	64 ± 1	103 ± 3
*R*_g_ from Guinier (Å)	69.9 ± 0.3	55.9 ± 0.3	16.3 ± 0.03	25.3 ± 0.6
*qR*_g_	1.18	1.04	1.28	1.06
Porod volume (10^3^)	3070.9	228.0	131.7	921.9
*I*(0)	0.269	0.459	0.013	0.079
*R*_g_ from *P*(*r*) (Å)	70.0	53.8	16.9	24.9
χ^2^ of fit	6.96	17.3	1.43	3.39
*CRYSOL* fit	173.5	381.5	4.97	2.86

## Data Availability

The genome sequence of phage Carin-1 has been deposited in GenBank under accession No. PQ119497.1. The coordinates of the crystal structures have been deposited in the Protein Data Bank with accession codes 28lx, 28mg and 28nb. The small-angle X-ray scattering data have been deposited in SASBDB with accession Nos. SASDYX6 (Dpo31-ΔD1), SASDYY6 (D4) and SASDYZ6 (Dpo31-wt).
