# Supplementary material for: Biochemical and structural characterization of a tail-spike protein with depolymerase activity identified in a marine podovirus
Source: Acta Crystallogr D Struct Biol. 2026 Jun 17;82(Pt 7):785–99. doi: 10.1107/S2059798326005425 (PMC13317679; doi:10.1107/S2059798326005425)
Supplement: Supplementary file 1 [file d-82-00785-sup1.pdf]

Acta Cryst D

STRUCTURAL BIOLOGY

Volume 82 (2026)

Supporting information for article:

Biochemical and structural characterization of a tail-spike protein with depolymerase activity identified in a marine podovirus

Serena Sirigu, Thomas Roret, Pierre-Yves Mocaër, Robert Larocque, Diane Jouanneau, Pierre Legrand, Anne-Claire Baudoux and Mirjam Czjzek

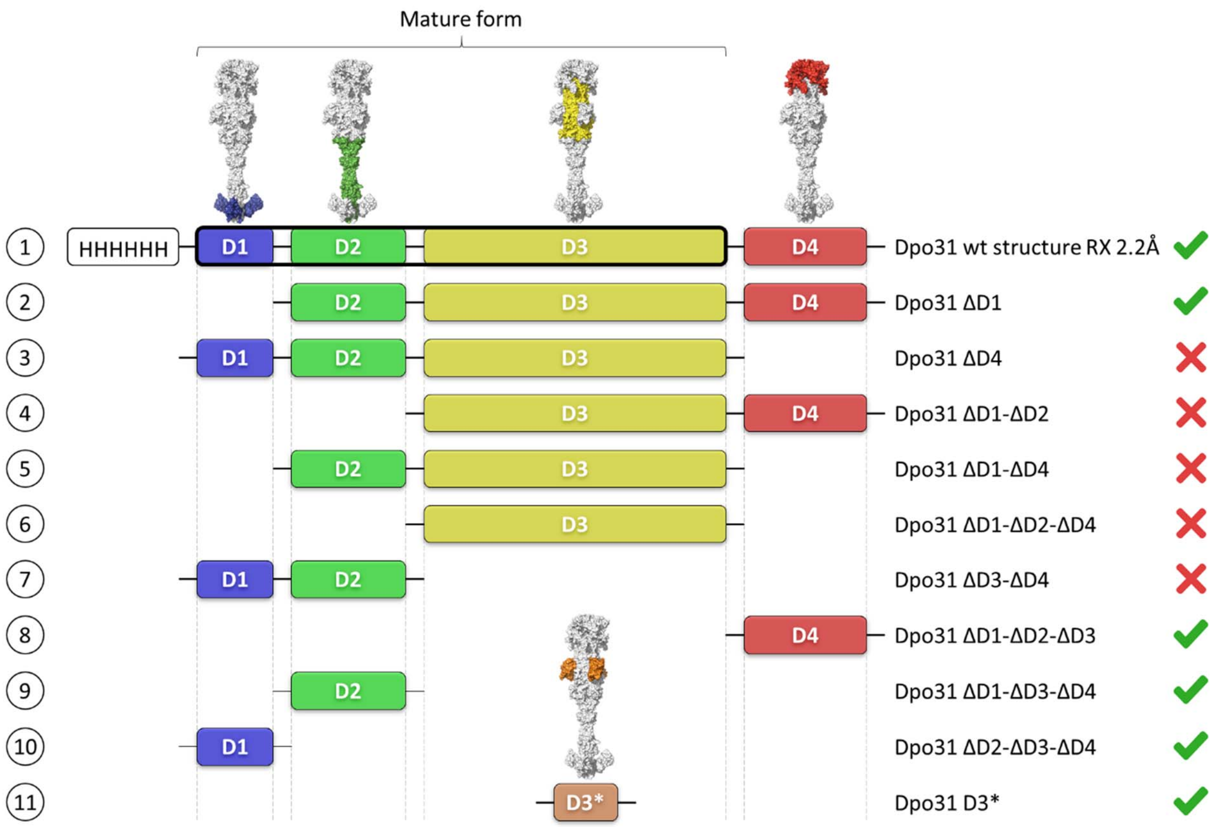

Figure S1 Domain organization and construct design of Dpo31. Schematic representation of the different Dpo31 constructs generated for expression and solubility testing. Constructs yielding soluble protein are indicated with green labels, while those that were insoluble are marked in red. The black frame highlights domains D1–D3 of the full-length protein, which are resolved in the crystal structure. Primary sequence of Dpo31 with the chosen domain boundaries for the different constructs are colored and delimitated as follows; domain D1 (blue): Met1–Tyr102; domain D2 (green): Pro103–Ser256; domain D3 (yellow): Ser257–Ala663; D3*: Gly434–Gly516; domain D4 (red): Asp664–Lys828.

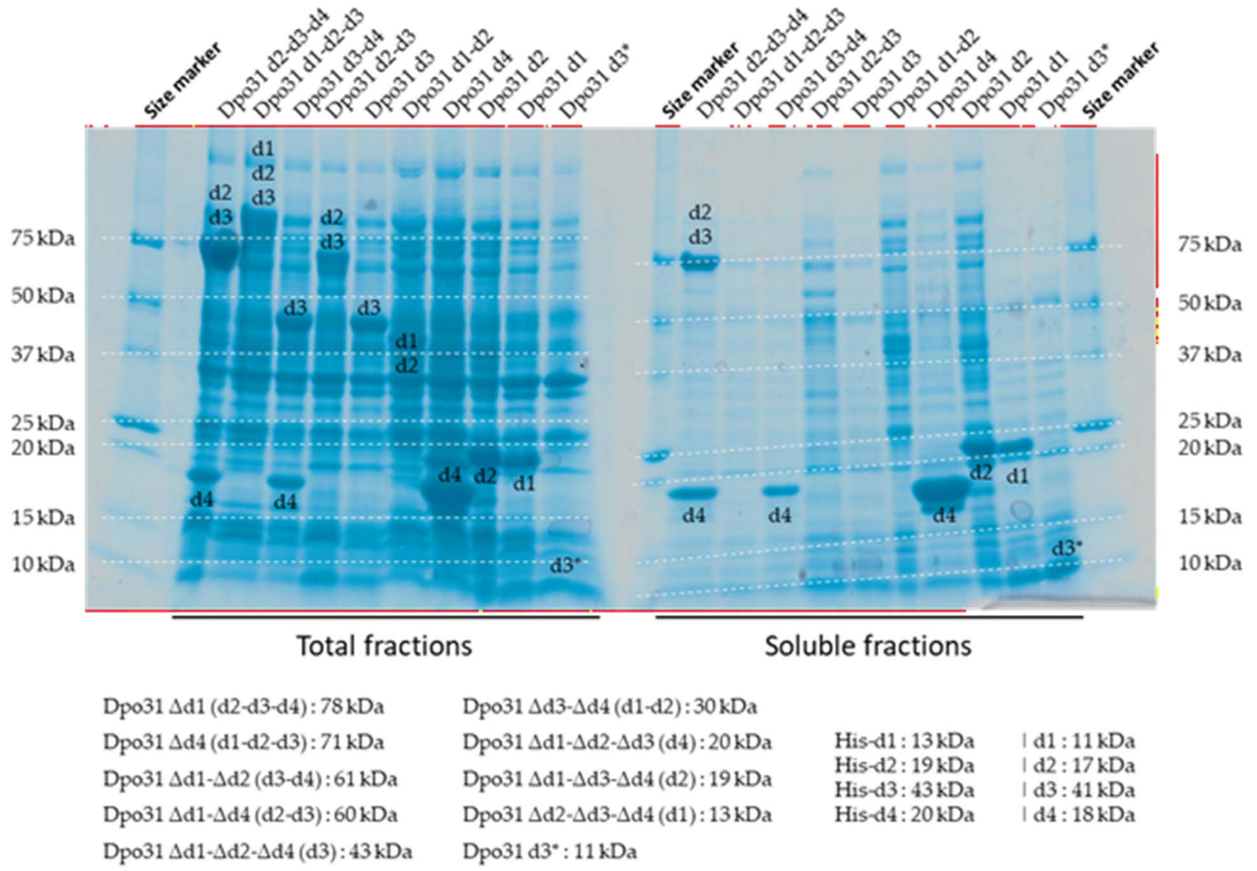

Figure S2 SDS–PAGE analysis of Dpo31 constructs. Coomassie-stained SDS–PAGE gels (reducing conditions) showing the purified Dpo31 constructs; molecular-weight markers (M) and construct identities (D1, D2, D3, D3*, D4 and combinations) are indicated on the gels. Dashed lines mark approximate band positions to ease comparison between lanes. The labels above the lanes describe the respective constructs that were cloned and not necessarily the protein that is expressed. A recurrent band at ~18 kDa (arrow) is visible in all lanes derived from constructs that include D4, consistent with proteolytic cleavage of D4 and accumulation of the isolated D4 fragment. These results indicate that when D4 is present in the expression construct it is cleaved off, and the mature form lacking D4 is the predominant species observed. Band assignments are based on the expected molecular weights of the domain constructs.

A)

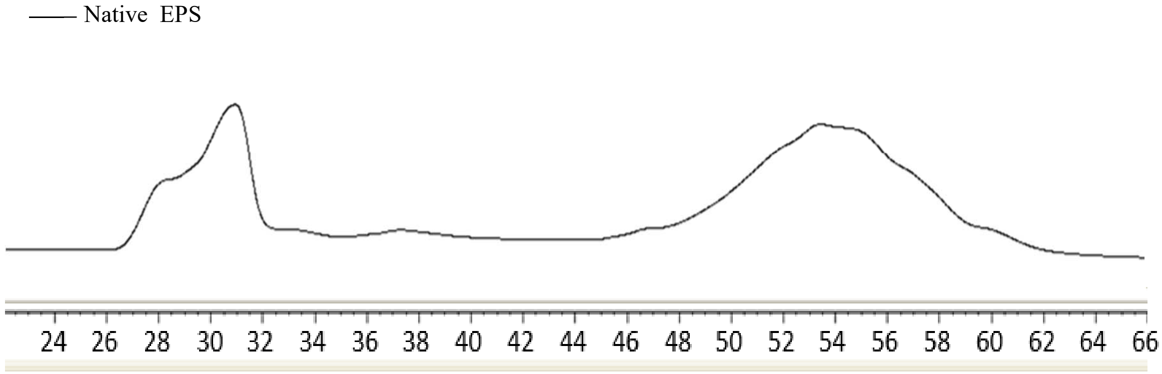

B)

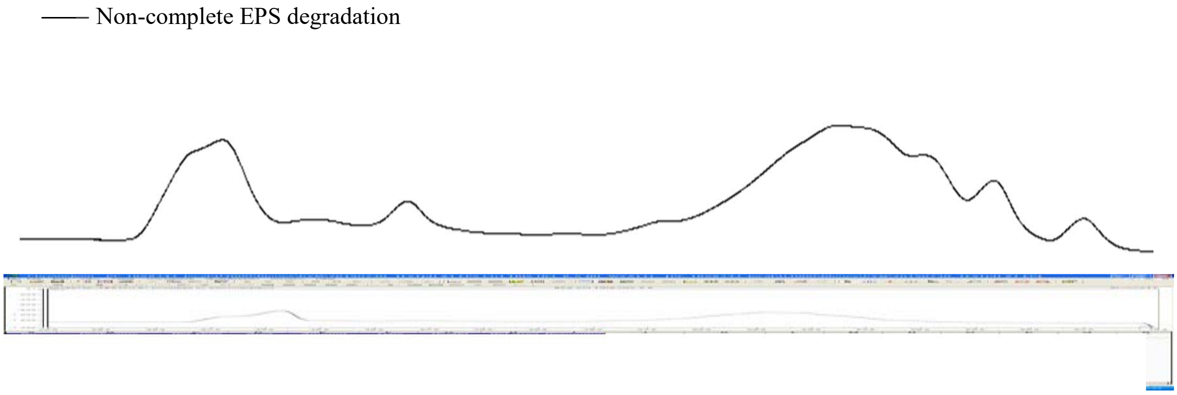

C)

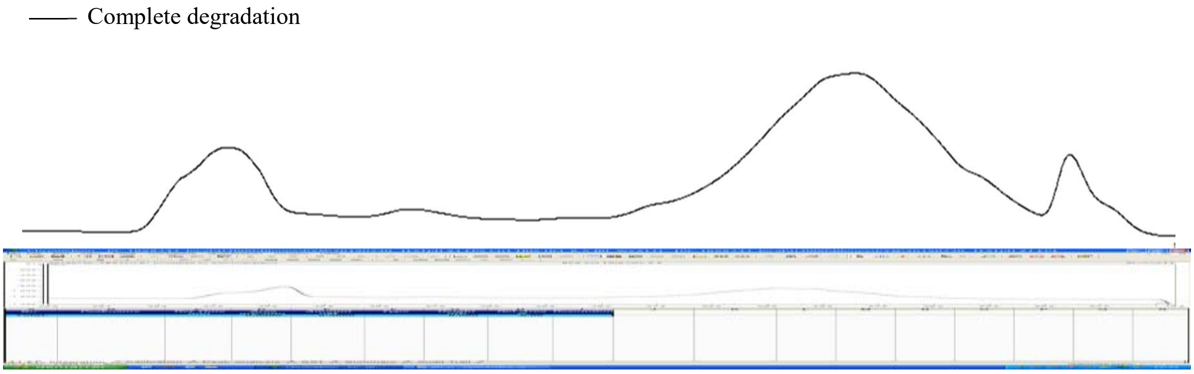

Figure S3 SEC-RI analysis of EPS degradation by Dpo31. Degradation of native EPS (A), non-complete degraded EPS (B), and completely degraded EPS (C). Refractory Index is shown against time (in minutes)

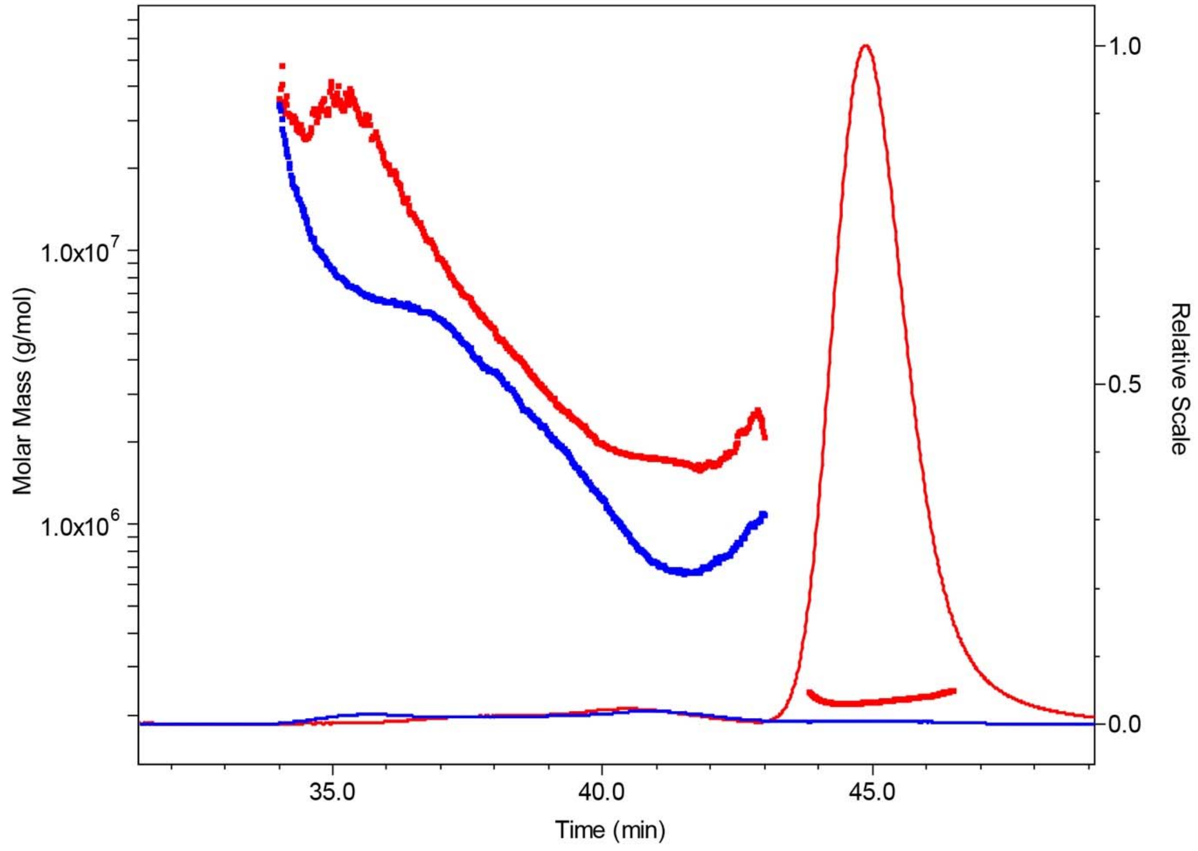

Figure S4 HPSEC-MALS analysis of EPS degradation by Dpo31. The undegraded EPS, or negative control, is shown in blue, and the EPS after degradation by Dpo31 is shown in red. The thick lines represent the molar mass and the thin line represent the chromatogram given by the refractive index signal. The undegraded EPS is constituted only of large molecules, eluting early in the chromatogram (before 43 min), while the degraded EPS shows an additional well-defined peak around 45 min, of smaller molecular weight.

Table S1 Molecular weights, calculated by HPSEC-MALS, of the EPS before (native) and after degradation by Dpo31.

	**Native EPS**		**EPS degraded by Dpo31**	
	**Mw (g/mol)**	**Polydispersity**	**Mw (g/mol)**	**Polydispersity**
**Peak 1**<br>34.0-43.0 min	3.446e+6	2.337	4.562e+6	1.813
**Peak 2**<br>43.8-48.5 min	absent	absent	2.254e+5	1.001

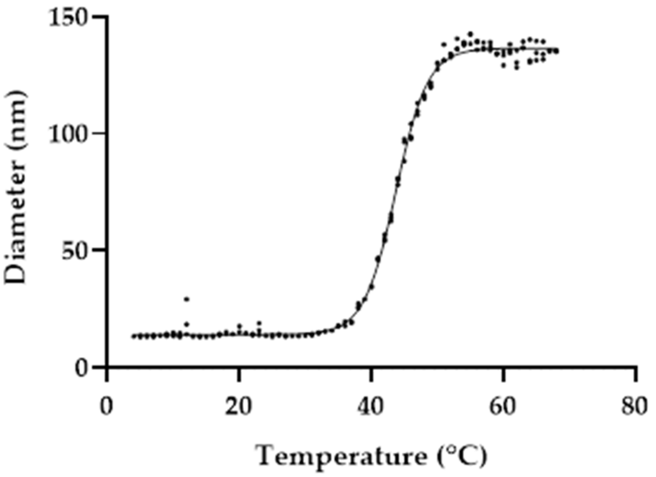

Figure S5 Thermal denaturation of the CARIN1 phage polysaccharidase measured by DLS. Hydrodynamic diameter (nm) as a function of temperature (°C) measured by dynamic light scattering (DLS). Measurements were performed on 30 µl samples of protein at 12.18 mg·ml^{-1} over the temperature range 4-68 °C with 1 °C increments; each temperature point is the mean of three independent measurements (n = 3). The curve shows the thermal transition of the enzyme and a sigmoidal fit to the averaged data. The melting temperature (Tm), defined as the midpoint of the transition, is 43.72 ± 0.15 °C.

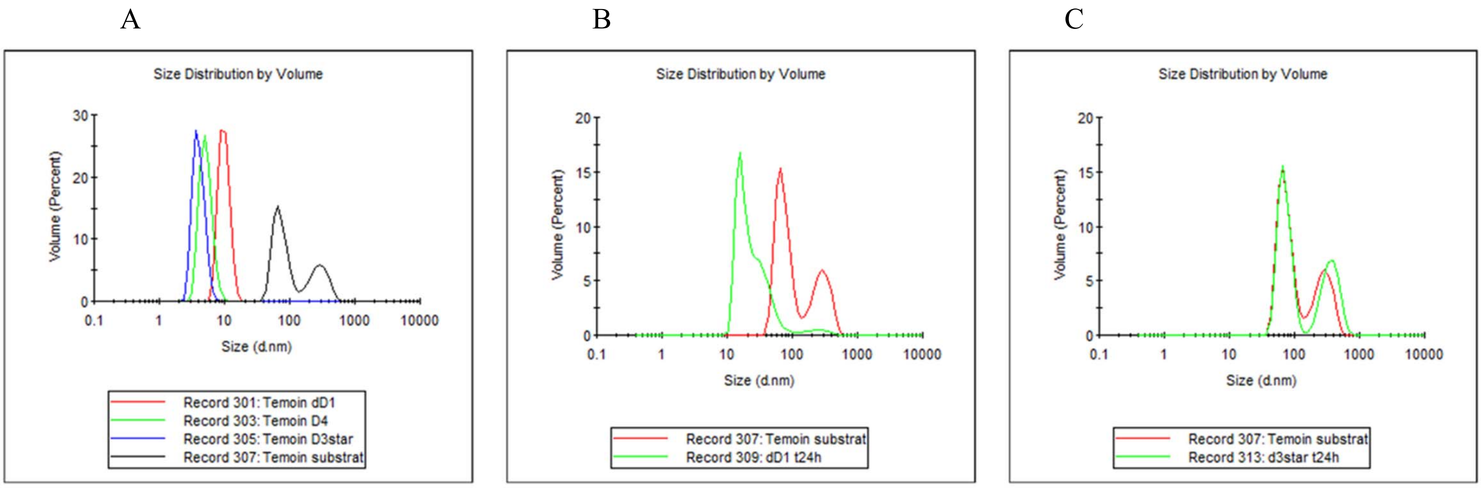

Figure S6 DLS measurements of EPS degradation by Dpo31-ΔD1, D3* and D4. (A) Superimposition of DLS signals of proteins alone: D3*(blue), D4 (green) and Dpo31-ΔD1 (red) and substrate alone (black). (B) DLS signal of substrate without Dpo31-ΔD1 (red) and after incubation with Dpo31-ΔD1 (green) showing a clear decrease in the average hydrodynamic radius of the molecules in solution. (C) DLS signal of substrate without (red) and with D3* (green) showing inactivity of this domain and no decrease of the average hydrodynamic radius of the molecules in solution.

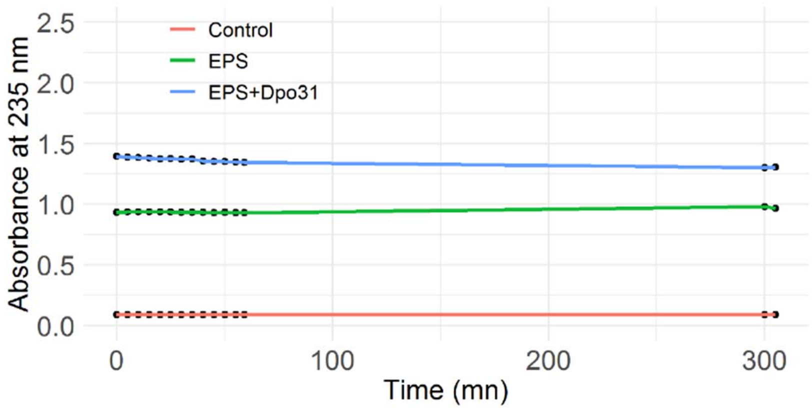

Figure S7 Monitoring Lyase activity of Dpo31. The degradation by lytic activity can be monitored by absorbance at 235 nm. The figure shows the measurements accumulated during 300 minutes (5 hours). The complete absence of signal indicates that Dpo31 is not a lyase.
